# Supplementary material for: Reference-guided assembly of metagenomes with MetaCompass
Source: Cell Rep Methods. 2025 Sep 26;5(10):101186. doi: 10.1016/j.crmeth.2025.101186 (PMC12570322; doi:10.1016/j.crmeth.2025.101186)
Supplement: Document S1. Figures S1–S9 and Tables S2–S4 [file mmc1.pdf]

Cell Reports Methods, Volume *5*

Supplemental information

Reference-guided assembly of metagenomes with MetaCompass

Tu Luan, Victoria P. Cepeda-Espinoza, Bo Liu, Zac Bowen, Ujjwal Ayyangar, Mathieu Almeida, Sergey Koren, Todd J. Treangen, Adam Porter, and Mihai Pop

SUPPLEMENTARY MATERIAL

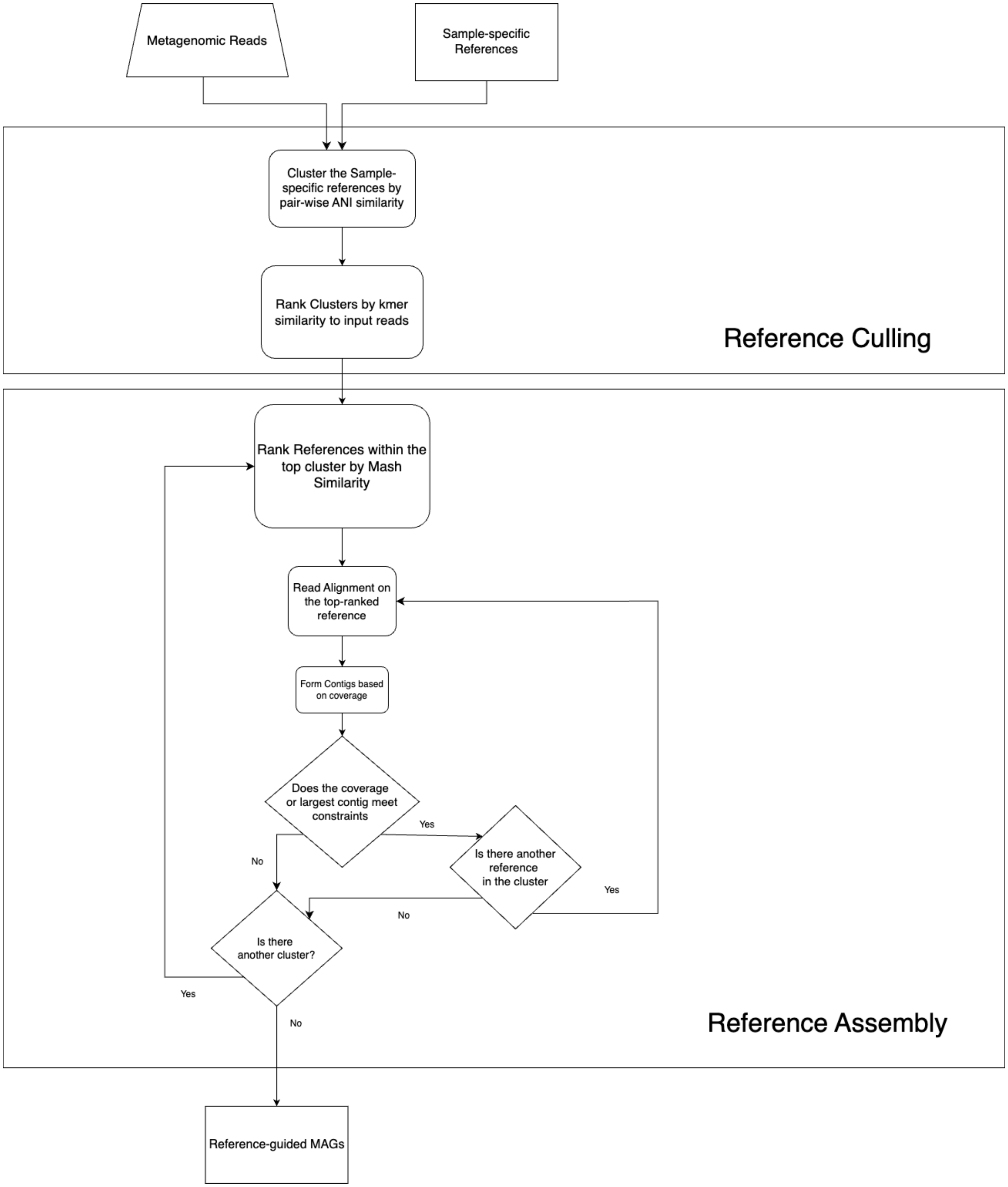

Supplementary Figure S1. The workflow of Reference Selection and Reference Assembly, related to Figure 1.

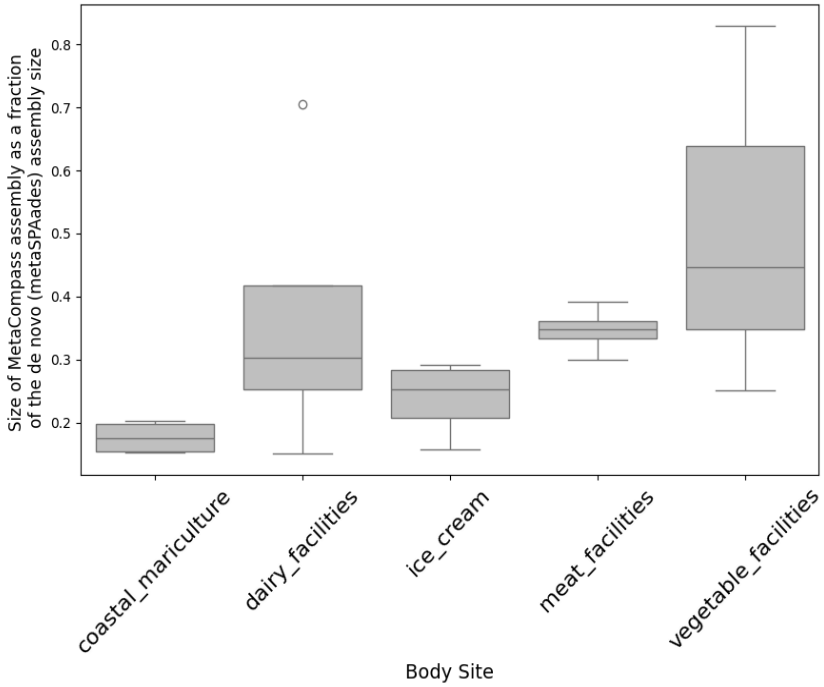

Supplementary Figure S2. Fraction of the de novo assembly that is captured by the reference-guided assembly for non-human samples, related to Figure 2. The numbers are notably lower than those for human-associated microbiome samples, reflecting the poorer representation of environmental microbes in public databases.

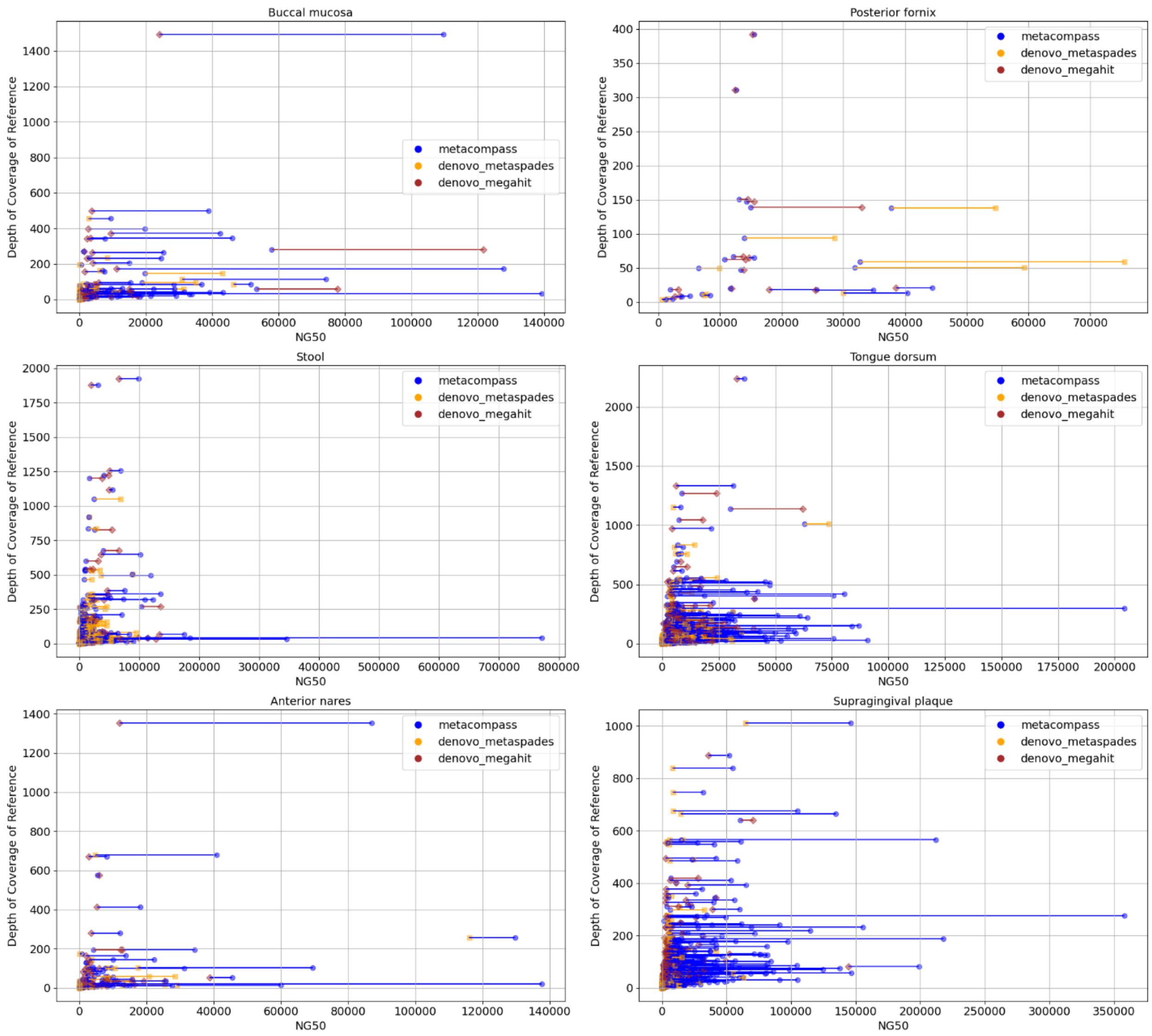

Supplementary Figure S3. Comparison between MetaCompass and *de novo* assembly methods on the measurement of NG50 of the clusters versus the depth of coverage of references, related to Figure 3. Data are shown for 15 stool samples from each body site. The length of the line connecting the two assembly NG50 points represents the difference between the NG50 values of the two points.

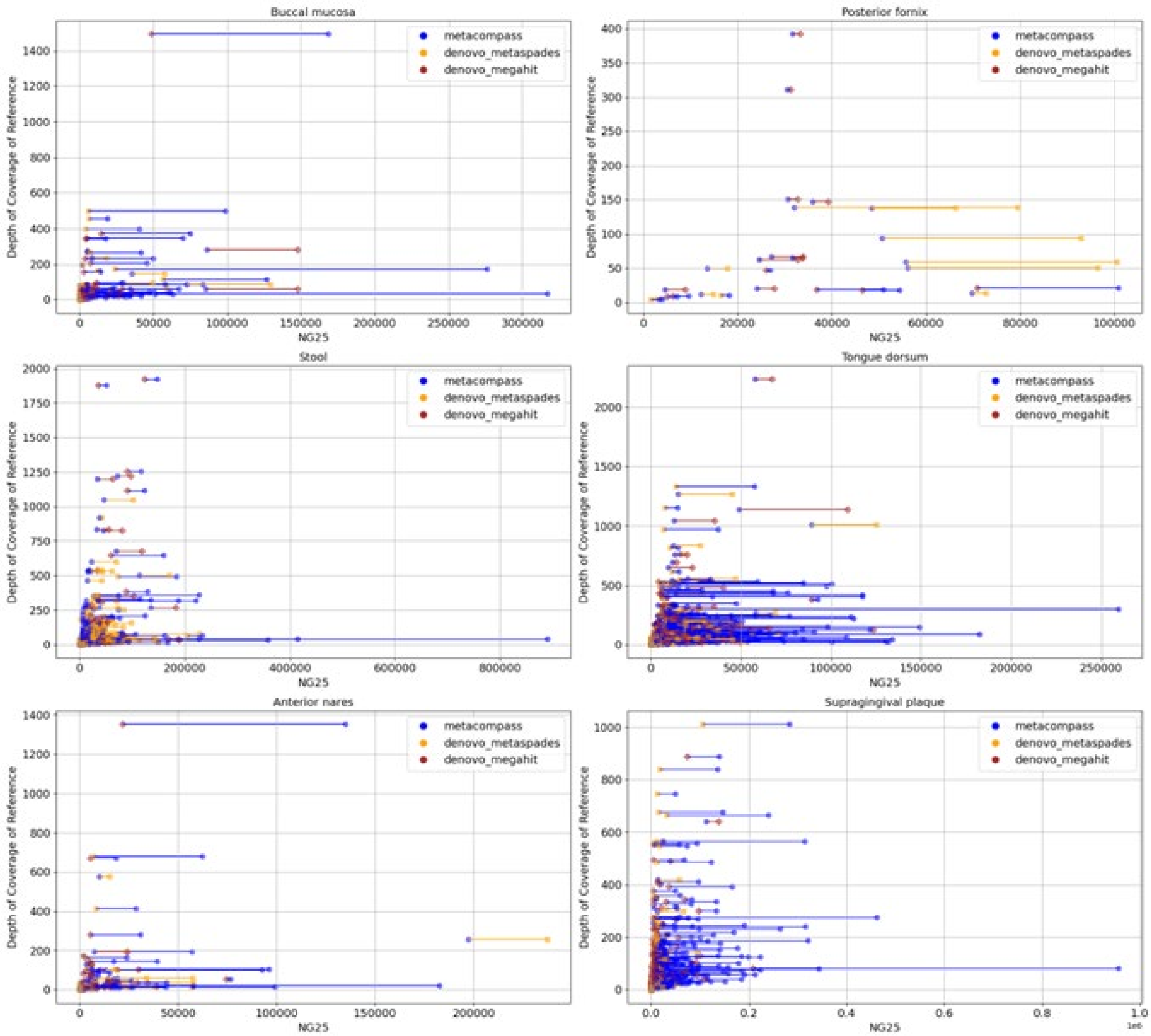

Supplementary Figure S4. Comparison between MetaCompass and *de novo* assembly methods on the measurement of NG25 of the clusters versus the depth of coverage of references, related to Figure 3. Data are shown for 15 samples from each body site. The length of the line connecting the two assembly NG25 points represents the difference between the NG25 values of the two points.

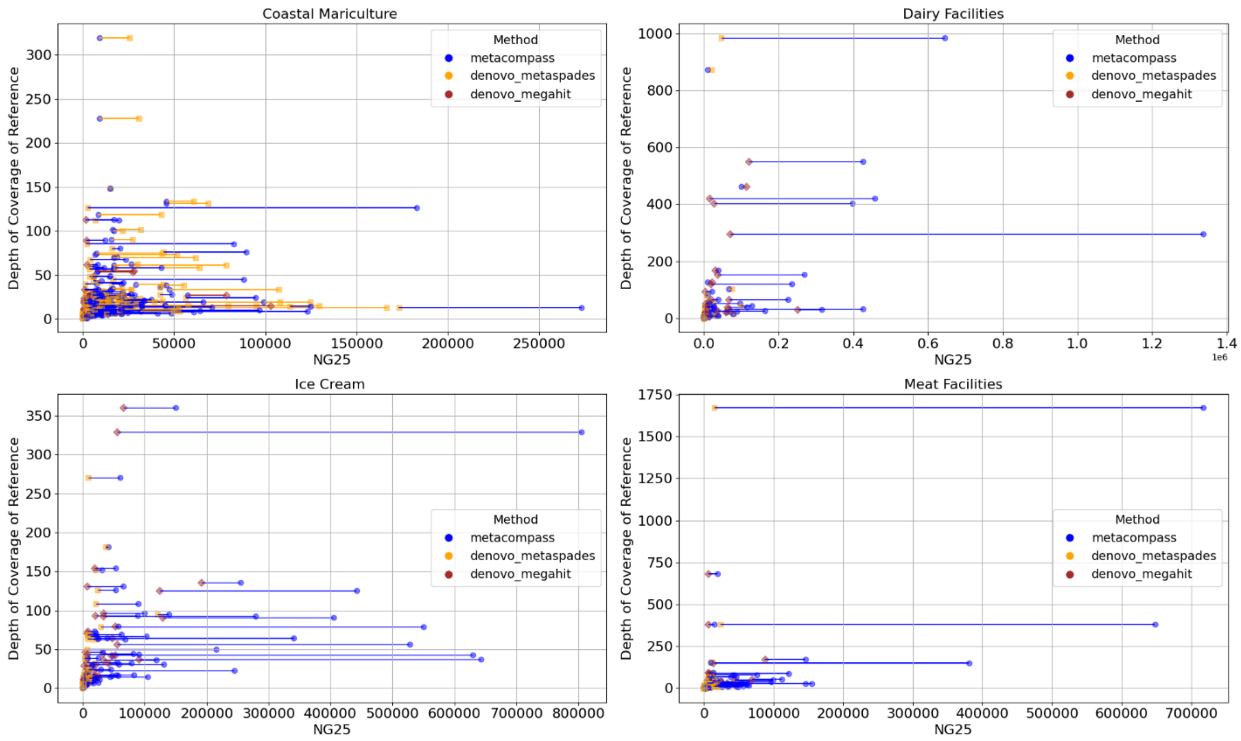

Supplementary Figure S5. Comparison between MetaCompass and *de novo* assembly methods on the measurement of NG50 of the clusters versus the depth of coverage of references for non-human sites, related to Figure 3. The four panels represent: a coastal mariculture system, and sampling of an ice cream manufacturing facility and meat and dairy facilities. The length of the line connecting the two assembly NG25 points represents the difference between the NG25 values of the two points.

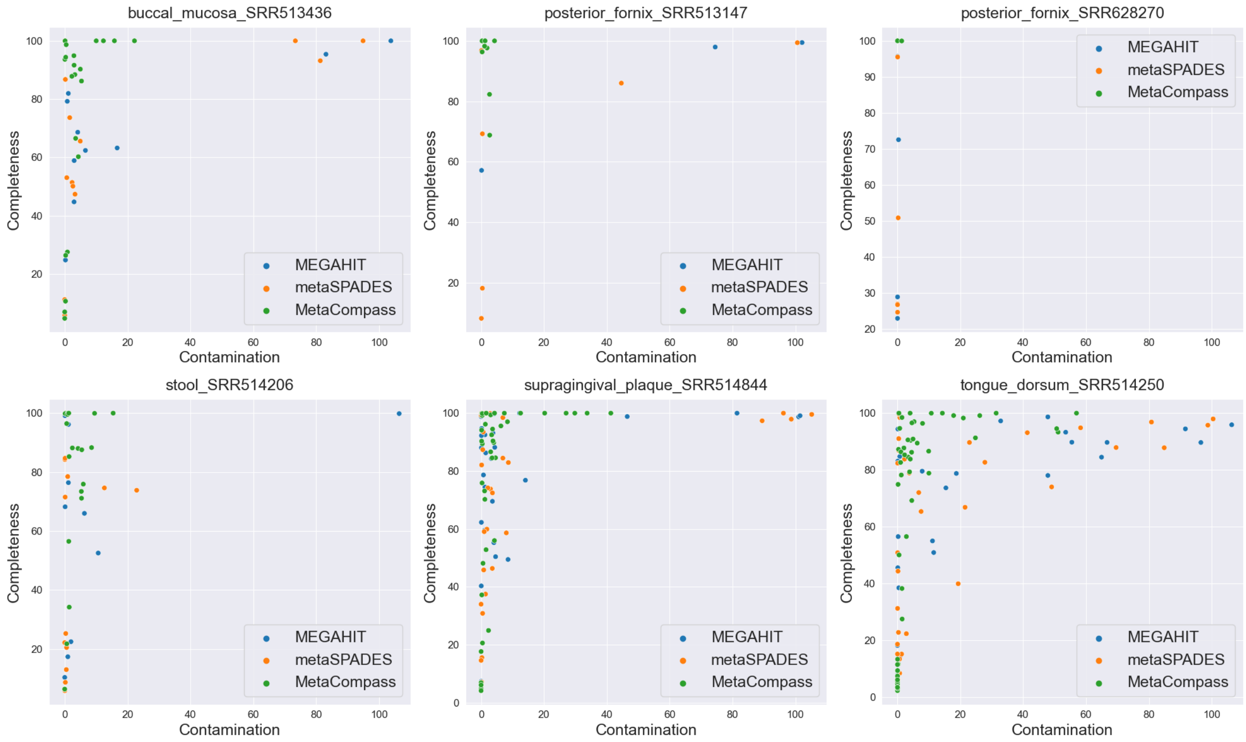

Supplementary Figure S6. Comparison between MetaCompass and *de novo* assembly methods based on completeness and contamination levels for genome-level bins, related to Table 1. Data are shown for six samples from different body sites. MetaCompass bins are defined by the assemblies generated by individual reference genome clusters. *De novo* bins are generated using MetaBat2.

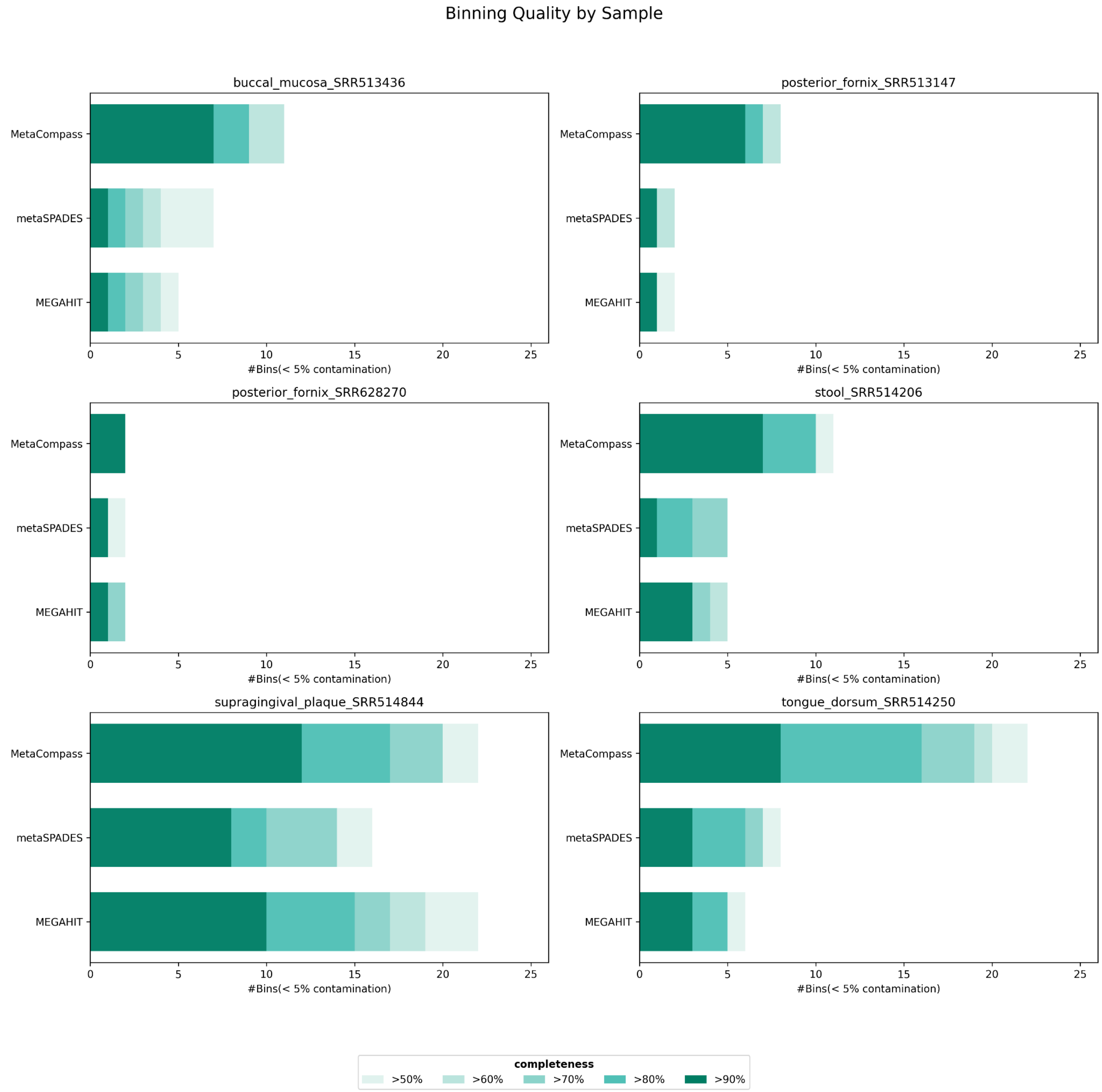

Supplementary Figure S7. Binning completeness for a fixed level of contamination, related to Table 1. A larger number of MetaCompass genome-level bins has a high level of completeness compared to de novo methods, looking only at the bins with low level of contamination.

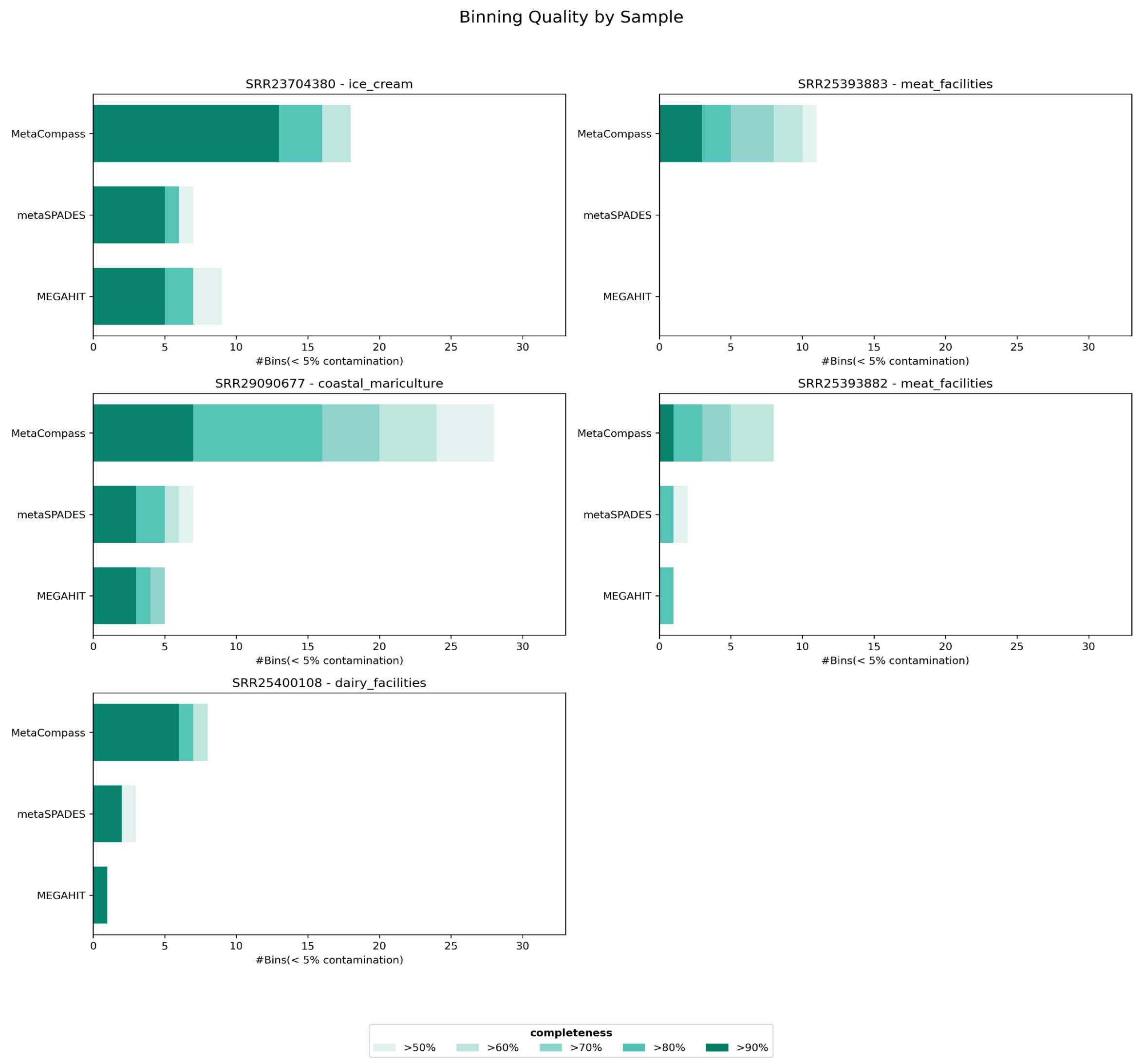

Supplementary Figure S8. Binning completeness versus contamination for non-human microbiome samples, related to Table 1. A larger number of MetaCompass genome-level bins has a high level of completeness compared to de novo methods, looking only at the bins with low level of contamination.

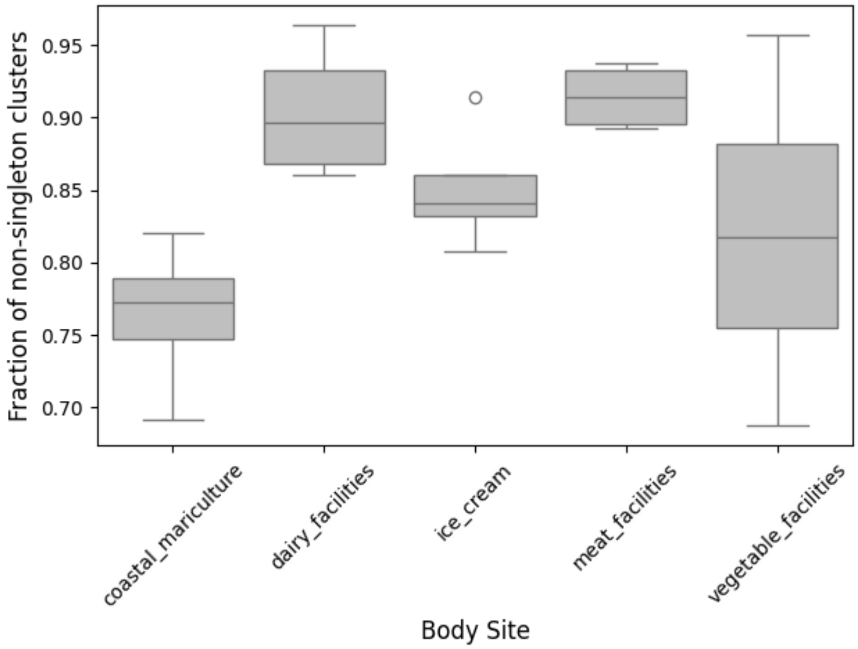

Supplementary Figure S9. Distribution of non-singleton clusters for non-human samples, related to Figure 4. The overall fraction of non-singleton clusters appears slightly higher than for human samples.

Supplementary tables

Supplementary Table S2. Effects of sequencing coverage depth on the reference-guided assembly, Related to STAR Methods. Buccal mucosa sample SRR513142 was analyzed at different levels of rarefaction.

Fraction sub-sampled	Number of reads used by MetaCompass	Number of bases assembled	Average depth of coverage	Total number of marker genes covered in reference selection	Number of reference genomes used
5%	0	0	0	1,470	0
10%	236,390	3,461,370	6.7	1,950	3
20%	704,311	5,682,069	12.2	2,744	4
40%	2,348,667	14,840,897	15.5	3,460	12
60%	3,938,244	22,776,298	17.0	3,846	19
80%	5,436,057	26,890,252	19.8	4,075	22
100%	7,024,805	31,400,140	21.9	4,315	25

Supplementary Table S3. Comparison of assembly statistics between MetaCompass with default database and reduced database, related to STAR Methods.

Body site	Sample	Assembled length using default database (Mbp)	Maximum contig length using default database (kbp)	Assembled length using reduced database (Mbp)	Maximum contig length using reduced database (kbp)
tongue dorsum	SRR514250	95.6	271.3	57.7	180.1
buccal mucosa	SRR513436	38.4	264.4	41.8	127.3
posterior fornix	SRR513147	13.0	159.7	8.8	376.6
posterior fornix	SRR628270	3.4	165.3	6.2	183.3
supragingival plaque	SRR514844	85.4	420.4	70.0	325.6
stool	SRR514206	63.7	406.9	29.1	149.9

Supplementary Table S4. Comparison of runtime and assembly statistics for MetaCompass using the default and reduced databases, related to Table 2. Runtime was evaluated on a Linux 16-core (3.0GHz AMD® EPYC® 7313) server with 256 GB memory.

Body site	Sample	Number of reads	Sample richness	# genomes assembled default database	# genomes assembled reduced database	Runtime default database	Runtime reduced database
tongue dorsum	SRR514250	226,602,332	133	211	36	16:29:48	05:24:49
buccal mucosa	SRR513436	33,183,142	154	52	38	01:09:50	01:15:52
posterior fornix	SRR513147	7,038,812	30	32	6	00:21:02	00:07:48
posterior fornix	SRR628270	2,713,906	12	4	5	00:07:02	00:03:31
supragingival plaque	SRR514844	112,950,078	126	115	45	06:50:27	03:16:16
stool	SRR514206	171,825,210	48	104	14	07:56:40	02:37:06
