# Supplementary material for: Reference-guided assembly of metagenomes with MetaCompass
Source: Cell Rep Methods. 2025 Sep 26;5(10):101186. doi: 10.1016/j.crmeth.2025.101186 (PMC12570322; doi:10.1016/j.crmeth.2025.101186)
Supplement: Document S2. Article plus supplemental information [file mmc3.pdf]

Cell Reports
Methods

Article

Reference-guided assembly of metagenomes with MetaCompass

Graphical abstract

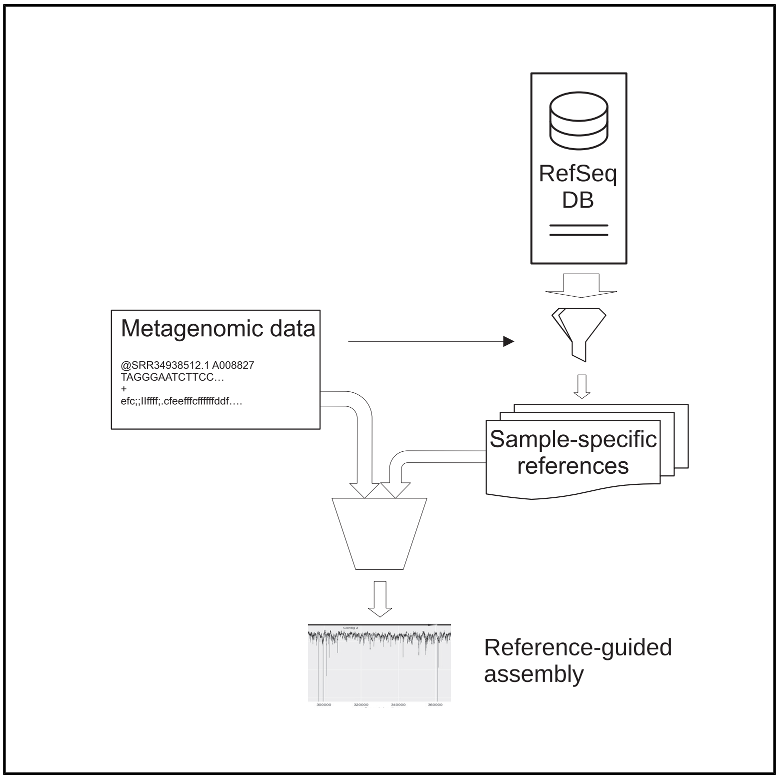

Highlights

- The use of reference sequences can improve genomic analyses
- Hundreds of thousands of bacterial genome reference sequences are available
- Gene-based indexing effectively selects sample-specific reference genome sequences
- Reference-guided assembly of metagenomic data improves and augments *de novo* methods

Authors

Tu Luan, Victoria P. Cepeda-Espinoza, Bo Liu, ..., Todd J. Treangen, Adam Porter, Mihai Pop

Correspondence

mpop@umd.edu

In brief

Reference-guided methods are common in human genomics but not used to analyze microbial community data. Luan et al. describe an approach for reference-guided assembly of microbiome samples and identifying which genomes among the many in public databases can be effectively used to guide the analysis.

Luan et al., 2025, Cell Reports Methods *5*, 101186
October 20, 2025 © 2025 The Author(s). Published by Elsevier Inc.
https://doi.org/10.1016/j.crmeth.2025.101186

CellPress

Cell Reports Methods

CellPress OPEN ACCESS

Article

Reference-guided assembly of metagenomes with MetaCompass

Tu Luan,[1,2,6] Victoria P. Cepeda-Espinoza,[1,2,6] Bo Liu,[1,2,6] Zac Bowen,[3] Ujjwal Ayyangar,[3] Mathieu Almeida,[2] Sergey Koren,[4] Todd J. Treangen,[2,5] Adam Porter,[1] and Mihai Pop[1,2,7,*]

[1]Department of Computer Science, University of Maryland, College Park, MD 20742, USA

[2]Center for Bioinformatics and Computational Biology, University of Maryland, College Park, MD 20742, USA

[3]Fraunhofer USA Center Mid-Atlantic, Riverdale, MD 20737, USA

[4]Genome Informatics Section, Computational and Statistical Genomics Branch, National Human Genome Research Institute, Bethesda, MD 20892, USA

[5]Department of Computer Science, Rice University, Houston, TX 77005, USA

[6]These authors contributed equally

[7]Lead contact

*Correspondence: mpop@umd.edu

https://doi.org/10.1016/j.crmeth.2025.101186

MOTIVATION The number of complete bacterial genome sequences available in public databases is rapidly increasing, but this resource has yet to be effectively used in guiding the analysis of microbiome samples. We aimed to develop an effective technique for using vast genome sequence collections to serve as a reference in the analysis of microbiome data, addressing the challenge of determining which, among the hundreds of thousands of genome sequence that are publicly available, can be used as references for guiding the analysis of a given sample.

SUMMARY

Metagenomic studies have primarily relied on *de novo* assembly for reconstructing genes and genomes from microbial mixtures. While reference-guided approaches have been employed in the assembly of single organisms, they have not been used in a metagenomic context. Here, we develop an effective approach for reference-guided metagenomic assembly that can complement and improve upon *de novo* metagenomic assembly methods for certain organisms. Such approaches will be increasingly useful as more genomes are sequenced and made publicly available.

INTRODUCTION

Microorganisms play an important role in virtually all of the Earth's ecosystems and are critical for the health of humans,[1] plants, and animals. Most microbes, however, cannot be easily grown in a laboratory.[2] The analysis of organismal DNA sequences obtained directly from an environmental sample (a field termed metagenomics), enables the study of microorganisms that are not easily cultured. Metagenomic studies have exploded in recent years due to the increased availability of inexpensive high-throughput sequencing technologies. Some examples include the MetaHIT[3] and Tara Ocean projects[4] in Europe, the Human Microbiome Project (HMP) in the US,[5] as well as crowd-sourced projects such as American Gut.[6]

The analysis of these vast amounts of data is complicated by the fact that reconstructing large genomic segments from metagenomic reads is a formidable computational challenge. Even for single organisms, the assembly of genome sequences from short reads is a complex task, primarily due to ambiguities in the reconstruction that are caused by genomic repeats.[7] In addition, metagenomic assemblers must tolerate the non-uniform representation of genomes in a sample as well as genomic variants between the sequences of closely related organisms. Despite advances in metagenomic assembly algorithms over the past years,[8–13] the computational difficulty of the assembly process remains high, and the quality of the resulting assemblies requires improvement.

Consequently, many analyses of metagenomic data are performed directly on unassembled reads[14–18]; however, the much shorter genomic context leads to lower accuracy.[19] The relatively recent advancement of long read assembly technology[20,21] has mitigated the challenges posed by repeats[22–24]; however, the need for effective and efficient metagenomic assembly approaches remains high, particularly since long read technologies are not yet widely used in metagenomic applications due to lower throughput, higher costs, and higher required DNA quality and concentration.[25,26]

Reference-guided, comparative assembly approaches have been used previously to assist with the assembly of short reads

© 2025 The Author(s). Published by Elsevier Inc.
This is an open access article under the CC BY license (http://creativecommons.org/licenses/by/4.0/).

when a closely related reference genome was available.[27,28] Such approaches work as follows. Short sequencing reads are aligned to a reference genome of a closely related species, and then their reconstruction into contigs is inferred from their relative locations in the reference genome.[28] This process overcomes, in part, the challenge posed by repeats, as the entire read (not just the segment that overlaps within adjacent reads) provides information about its location in the genome.

To date, hundreds of thousands of bacterial genomes have been sequenced to a high level of quality,[29] and this number is expected to grow rapidly thanks to long read technologies. These sequenced genomes are a great resource for performing reference-guided assembly of metagenomic sequences. Techniques developed in the context of single genomes cannot, however, be used directly in a metagenomic setting. Simply mapping a set of reads to even hundreds of different genomes is currently computationally prohibitive. Furthermore, genome databases comprise many variants of the same genome (e.g., the US Food and Drug Administration's GenomeTrackr project[30] alone has contributed over 500,000 different strains of *Salmonella*), and genome-by-genome analyses would result in redundant reconstructions of metagenomic sequences. We also note that some recent reference-guided strategies implemented in genomic analysis tools, such as the "–trusted-contigs" feature of the SPAdes assembler[31–33] and StrainPhlan,[34] ignore the fact that the data being reconstructed originate from genomes that are related but different from the genomes found in public databases. As a result, such approaches may mis-assemble the metagenomic data exactly within the genomic regions where novel biological signals may be located.

In this paper, we describe an effective assembly software package for the reference-based assembly of metagenomic data. We rely on an indexing strategy to quickly construct sample-specific reference collections, thereby dramatically reducing the computational costs of mapping metagenomic reads to all references. We align reads against closely related genomes only once, then follow with a polishing step to resolve the discrepancies between the metagenomic data and the reference genomes. We show that our reference-based assembly approach yields high-quality assemblies that generally outperform corresponding *de novo* assemblies of the same data without introducing significant computational overhead.

RESULTS

Introduction

To provide context to the results presented below, we briefly describe the strategy employed by MetaCompass to assemble metagenomic samples. Full details are provided in the STAR Methods section. MetaCompass starts with an initial reference selection step, where the input reads are aligned to a database comprising marker gene sequences in order to identify which reference genome sequences may be useful as a guide for assembly. Only reference genomes that contain a sufficient number of marker genes covered by reads are used in further analysis (default cutoffs selected to match the expected coverage of marker genes at between 1- and 3-fold coverage). To account for strain variation, reference genome sequences are clustered together based on their min-hash similarity, and each cluster is processed separately. The clusters are analyzed to determine their k-mer overlap with the set of reads, prioritizing further analysis in the order of the strength of the match between each cluster and the set of reads. Within each cluster, the reference genome sequences are also processed in order of their overlap with the set of reads. Reads are aligned to each genome sequence, the alignment information is then processed to identify breaks in coverage and other signatures of differences between the reference genome sequence and the metagenomic data, and the sequence of the resulting contigs is then determined using a sequence-polishing algorithm. After processing the first genome sequence in each cluster, additional genome sequences are processed as long as they add sufficient information to the assembly (estimated based on the set of reads that can still be aligned to genomes in the cluster; see more details in STAR Methods). Thus, MetaCompass can "mix and match" segments from multiple reference genome sequences to generate a more complete picture of the pangenome of the organism represented within a genome cluster (an overview of the pipeline is provided in Figure 1 and is expanded in Figure S1).

Data sources and overall statistics

We assessed the performance of MetaCompass by analyzing 90 metagenomic samples from the HMP,[5] downloaded from the NCBI SRA database. These samples, 15 each from six different body sites—the tongue dorsum, buccal mucosa, posterior fornix, supragingival plaque, stool, and anterior nares—were randomly selected from the full HMP dataset with the intent of capturing microbial communities of varied diversity and sequencing depth (information about the samples is provided in Table S1). To generate a baseline, we also generated *de novo* assemblies for all samples using metaSPAdes[35] (v.3.15.5) and MEGAHIT (v.1.2.9),[36] arguably the most commonly used assembly tools in metagenomic experiments.

The amount of sequencing data varied depending on the body site, likely due to the different extent of human DNA contamination in each sample. Stool samples contained most sequencing reads (a median of 179.7M), followed by tongue dorsum (median 156.1M), supragingival plaque (median 117.6M), buccal mucosa (median 13.7M), posterior fornix (median 3M), and anterior nares (median 559K). Ten samples, nine from the anterior nares and one from buccal mucosa, could not be assembled by MetaCompass because none of the reference genomes were covered at sufficient depth. The results shown below refer to just the 80 samples that were assembled by MetaCompass.

Additionally, we benchmarked MetaCompass using several non-human datasets: a coastal mariculture system[37] and food processing facilities[38] (Table S1).

Overall differences between MetaCompass and *de novo* assemblies

We compared the overall output of MetaCompass with *de novo* assemblies of the same samples generated using metaSPAdes. We excluded from the analysis the 10 samples that could not be assembled by MetaCompass and two additional

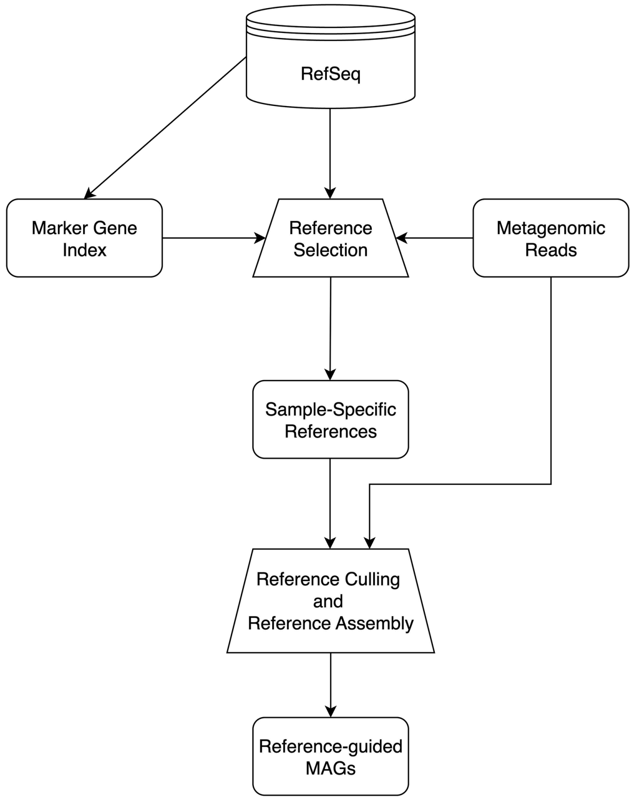

Figure 1. Overview of the MetaCompass pipeline

The MetaCompass pipeline begins with reference selection, where sample-specific reference genomes are identified based on marker gene coverage in the input reads. These genomes are clustered to remove redundancy and assembled in an iterative process based on the order of each cluster's similarity to the input read set. The detailed workflow of reference culling and assembly is presented in Figure S1. The final output of the MetaCompass pipeline is a set of metagenome-assembled genomes (MAGs).

samples (NCBI:SRR513776 and NCBI:SRR513777) that could not be assembled by metaSPAdes. In most cases, the total size of the MetaCompass assemblies was smaller than that produced by metaSPAdes, which was expected, since MetaCompass can only assemble the fraction of the metagenome that aligns to reference genome sequences. Nonetheless, the MetaCompass assembly represented up to 97% of the metaSPAdes assembly for a posterior fornix sample (NCBI:SRR513779), indicating that, in that sample, reference-guided assembly could be effectively used for the majority of the organisms in this sample. On average, the size of the MetaCompass assembly, as a fraction of total size of the *de novo* assembly, ranged from a median of 90% for the posterior fornix samples to a low of 31% for the buccal mucosa samples (see Figure 2 for the distribution of these fractions across body sites and Figure S2 for the distribution in non-human samples).

Contiguity of MetaCompass assemblies of individual genomes

We first explored whether MetaCompass provides a benefit over *de novo* assembly tools for the individual genomes selected as references. We compare the contiguity of the *de novo* and reference-guided assemblies, which were assembled by MetaCompass using its default database, for each genome cluster (MetaCompass clusters the reference genomes in order to reduce redundancy). To evaluate contiguity, we use the metric NG25,[39] defined as the size of the largest contig *c* so that the sum of the lengths of contigs longer than *c* exceeds 25% of the entire genome size. This measure is only well defined when the genome size is known, and we only apply it to assess the quality of the assembly within the context of individual reference genomes. We use the NG25 metric rather than the more commonly used NG50 in order to capture a larger fraction of the data; however, results for NG50 are reported in Figure S3. In the context of a cluster of closely related reference genomes, we use, as a baseline, the length of the longest genome in the cluster. Within each cluster, all contigs generated with the assistance of the references in the cluster are pooled together. We then use the same set of reads that was used by MetaCompass to generate *de novo* assemblies using metaSPAdes and MEGAHIT.

We focus our analysis on only the assemblies that cover a cumulative length of at least 25% of the reference genome's length. Figure 3 highlights the relationship between the NG25 values and the depth of coverage, estimated with respect to the longest reference genome sequence within the corresponding cluster. For clarity, for each assembly, we only highlight two values: the assembly that achieves the largest NG25 metric and the second-best assembly if MetaCompass is the "winner." If MetaCompass does not generate the most contiguous assembly, it is reported as the second measure irrespective of its relative rank among the three tools.

At smaller NG25 values and lower coverage depths (lower left corner, x<50,000 y<250), the differences between MetaCompass and the two *de novo* assemblers are small, and all methods are comparably likely to perform best for individual samples. Outside of this region, MetaCompass often outperforms the other two methods, as evidenced by more prevalent blue lines in Figure 3. Moreover, when MetaCompass outperforms a *de novo* assembly tool, it frequently does so by a large extent, as demonstrated by the longer lines with the rightmost endpoint corresponding to MetaCompass.

Breaking down the analysis by body site (Figure S4), we note that MetaCompass typically outperforms the *de novo* tools, except for the posterior fornix, where metaSPAdes performs the best. The organisms where metaSPAdes showed the greatest benefit over MetaCompass were *Bifidobacterium breve*, *Lactobacillus iners*, *Lactobacillus jensenii*, *Lactobacillus gasseri*, and *Lactobacillus crispatus*, all important members of the human vaginal microbiota. We examined the assemblies more closely to assess why MetaCompass was unable to produce large contigs and noted that the reference genomes differed sufficiently from the reads in the sample to prevent good read alignment, thus causing MetaCompass to break the assembly. These differences were apparent both in the alignment of reads to the reference genomes, highlighting multiple regions with substantially reduced depth of coverage, as well as in the alignment of the metaSPAdes contigs to the reference (data not shown).

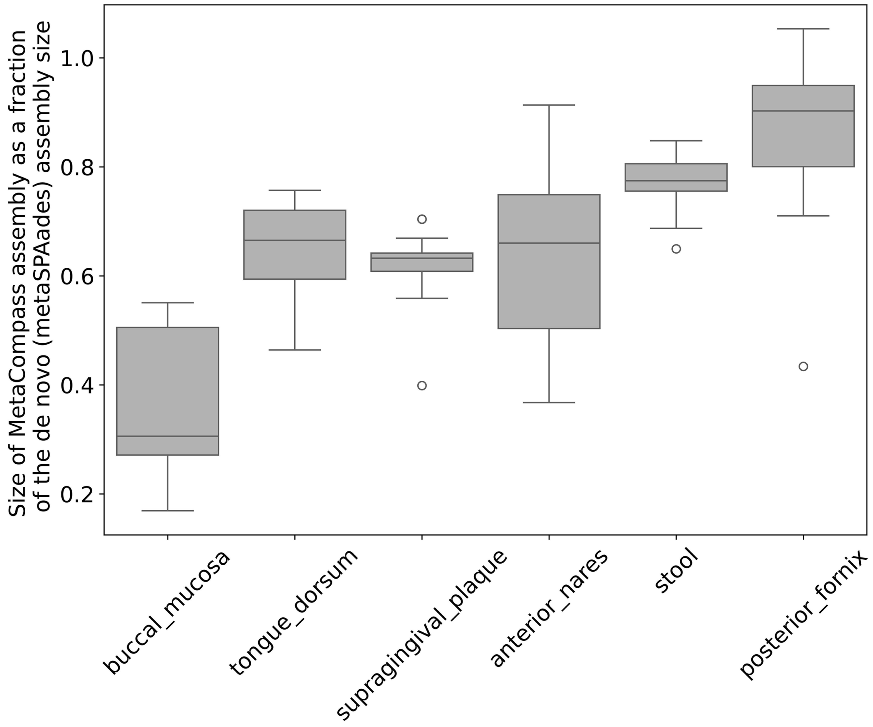

Figure 2. Comparing the total assembly size produced by MetaCompass to the *de novo* assembly size

Since MetaCompass can only assemble sequences that align to reference genomes, its assembly size reflects the fraction of the sample that can be "explained" by reference genome collections. The boxes represent the interquartile range (IQR: 25%–75%); whiskers are extending to the point that is furthest above/below the box that is within 1.5 IQR from the box. Circles represent outliers.

We also note that the overall contiguity of assemblies varied across body sites, with median NG25 values ranging from 10,182 for stool to 30,535 for the posterior fornix. This contiguity variation trend was also observed in the *de novo* assemblies, indicating that fundamental characteristics of the sample have a greater impact on contiguity than specific algorithmic choices.

We also benchmarked MetaCompass using environmental samples from coastal mariculture metagenomics studies and food processing metagenomics datasets (see data sources and overall statistics). The results (Figure S5) demonstrate a similar level of performance with respect to *de novo* methods as presented for human microbiome samples above. MetaCompass is least effective in the mariculture system (which is expected given the underexplored diversity of marine environments), though even in this setting it outperforms *de novo* methods for multiple organisms.

Quality of MetaCompass assemblies of individual genomes

Metagenomics-assembled contigs (MAGs) are usually assessed in terms of their completeness (estimate of how much of the genome is represented in the MAG) and contamination (the extent to which the MAG represents a mixture of two or more genomes). These measures are typically estimated based on a set of conserved marker genes. Here, we compare the output produced by MetaCompass to that generated using a MAG construction pipeline involving *de novo* assemblies (generated with MEGAHIT and metaSPAdes), which are then binned using MetaBAT2 (v.2:2.17). For MetaCompass, we use the "bins" represented by reference genome clusters, since they capture the pan-genome of organisms in a similar way as binning approaches. Completeness and contamination metrics for both reference-guided and *de novo* assemblies are computed with CheckM2 (v.1.0.1). As seen in Table 1, MetaCompass bins achieve higher completeness and lower contamination than *de novo* approaches (while the median contamination is higher for MetaCompass, the difference from *de novo* methods is insignificant, given that all methods have nearly 0 median contamination). MetaCompass achieves lower contamination levels across the whole range of completeness values even as contamination levels increase as more of the genomes are represented in the bins (Figure S6). This is also apparent when looking separately at just bins with low contamination levels (<5%), where a larger fraction of the MetaCompass assembly is contained in bins with high completion levels compared to MetaSpades and MEGAHIT (see Figure S7 for human samples and Figure S8 for non-human samples).

MetaCompass avoids reference bias

To evaluate whether the approach used by MetaCompass can account for large-scale differences between reads and reference genomes, we conducted a simulation to model situations where the reference genome contains genomic variants relative to the reads. The simulation was performed using sequencing reads from an isolate genome—*Escherichia coli* strain 2012C-4606 (BioSample:SAMN04498549), consisting of 1,833,258 paired-end reads— and the corresponding assembly, NCBI: GCA_003018795.1, which is 5.7 million bases long.

We simulated two types of differences between the reference genome and the reads in the sample: insertion and deletion events. To model insertions into the reference, we modified the largest contig from the assembly by inserting segments of random genomic alphabet in lengths of 125–1,250 bases, increasing in increments of 125 bases at a random location in the genome. These lengths were chosen to simulate insertion from half the read length to five times the read length, given that the read length of all reads is ~250 bases. We then used MetaCompass to assemble the sample using the modified genome sequence as a reference. For insertions larger than

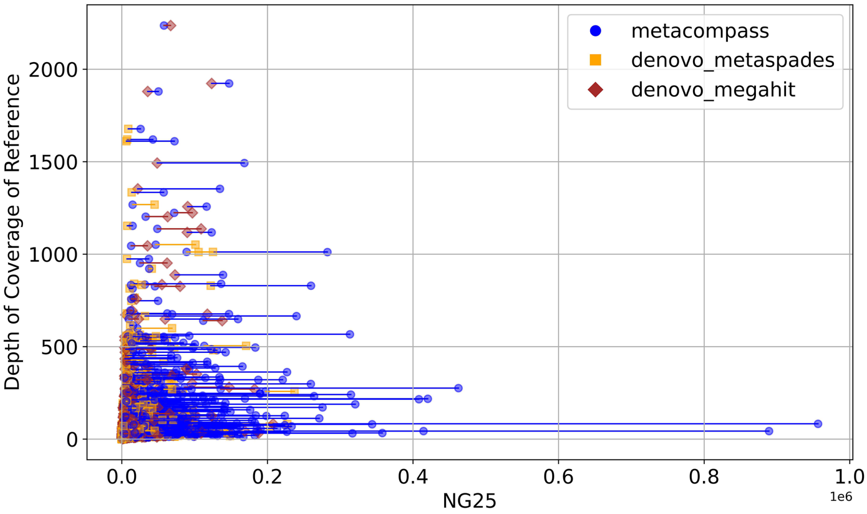

Figure 3. Comparison between MetaCompass and *de novo* assembly methods on the measurement of NG25 of the clusters vs. the depth of coverage of references from all samples

The length of the line connecting the two assembly NG25 points of the sample cluster represents the difference between the NG values of the two points.

125 bases, MetaCompass successfully detected a contiguity break at the insertion point and broke the assembly at that location. To model deletions, we removed genomic segments of lengths between 125 and 1,250 bases from the longest contig in the reference and performed reference-guided assembly using MetaCompass. MetaCompass was only able to detect a contiguity break for deletions longer than 875 bp. For all of the shorter deletion, the read alignment to the modified reference was able to span the breakpoint, thus obscuring the presence of a variant and allowing Pilon to generate an (incorrect) consensus sequence. This finding shows that further research is needed to identify small deletion events in order to avoid such misassemblies.

MetaCompass captures the pangenome of microbiome members

MetaCompass employs clustering as a key step in the analysis, grouping reference genomes into species-level clusters. Within these clusters, MetaCompass prioritizes reference genomes in descending order of k-mer similarity with the read set (as approximated by min-hashing distance). This strategy aims to ensure that the first genome assembled within a cluster is the one that is most similar to the corresponding genome within the sample. Reads that could not be aligned to the first reference genome are then iteratively aligned to the other reference genomes in the cluster, in decreasing order of their fit with the input data, with the intent of capturing genomic segments that were not present in the previously assembled genomes. This functionality can only be leveraged if the reference database captures strain-level diversity, as measured here by the number of reference genome clusters that contain two or more genomes. The proportion of non-singleton clusters varies across body sites (Figure 4, top) ranging from a high of 100% (all reference genome clusters comprise two or more reference genomes) for the posterior fornix to a low of 58.7% in supragingival plaques. We also observe a large extent of sample-to-sample variation in the ratio of non-singleton clusters captured within the anterior nares and buccal mucosa. Results for non-human samples are shown in Figure S9.

Within multi-genome clusters, MetaCompass frequently uses more than one genome (Figure 4, bottom), demonstrating that the genomes found in the samples do not necessarily have a good fit with any specific reference genome. At one extreme, in stool samples, MetaCompass used a median of 6 genomes per cluster, while in buccal mucosa and anterior nares, the median was close to 2.

To further demonstrate the use of multiple related reference sequences in the assembly process, we focus on cluster 11 from tongue dorsum sample NCBI:SRR514250. This cluster includes three reference genome sequences from the species *Streptococcus infantis*. The genome sequence of strain *S. infantis* STn450 showed the highest k-mer similarity to the reads and was assembled first. MetaCompass effectively assembled 1,548,950 bases, representing 84.57% of the reference genome. It generated 173 contigs larger than 2,000 bases, with the longest contig measuring 41,513 bases, and with an NG25 of 16,098. The second strain selected, *S. infantis* ATCC 700779, resulted in an assembly covering 599,811 bases (32.30% of the reference genome), with 38 contigs greater than 2,000 bases, the largest contig of 8,457 bases, and an NG25 of 625 bases. The assembly of the third genome, *S. infantis* NCTC13771, covered only 0.42% of the reference genome, with the longest contig of 2,121 bases and a total of 8,531 bases assembled, leading MetaCompass to terminate processing this cluster.

Another situation is represented by sample NCBI:SRR514223 from the buccal mucosa. Two nearly complete genomes are reconstructed from the same cluster, corresponding to two *Veillonella parvula* reference genomes. MetaCompass reconstructs 1.9M bp out of the 2M bp genome represented by accession NCBI:GCA_018370955.1_ASM1837095v1 and 1.7M bp out of the 3.3M bp genome represented by accession NCBI:GCA_003640195.1_ASM364019v1. This result is consistent with the previously characterized genomic diversity within the *Veillonella* genus[40] and demonstrates that MetaCompass is able to capture differences between closely related strains.

The effect of sequencing depth on the performance of MetaCompass

Metagenomic samples typically contain microorganisms with varying abundances, resulting in different sequencing depths. Additionally, the sequencing depth can vary based on

Table 1. Aggregate statistics for completeness and contamination for the genomic bins constructed with MetaCompass and with *de novo* approaches

Method	Mean completeness	Median completeness	Mean contamination	Median contamination
MetaCompass	**69.7**	**87.9**	**5.40**	1.5
MEGAHIT	66.6	78.7	17.7	1.1
MetaSPAdes	57.6	69.3	14.9	**0.7**

Bold indicates the best value (highest for completeness, lowest for contamination).

sample preparation, sequencing conditions, and equipment. To analyze the impact of depth of coverage on the effectiveness of MetaCompass, we focus on buccal mucosa sample NCBI:SRR513142, comprising 13,723,918 reads. We subsampled the data to 80%, 60%, 40%, 20%, 10%, and 5% of its original input size and processed these datasets using MetaCompass.

At full coverage, MetaCompass identified 25 reference genome sequences forming 24 clusters. As coverage was reduced, we noted a steady decline in the number of reference sequences selected for assembly, the total bases assembled, the average breadth and depth of coverage, and the number of marker genes covered in the reference selection process (Table S2). At 5% sampling rate, MetaCompass did not select any reference genomes, thus producing no assembly output.

MetaCompass achieves a high fraction of reads mapped

To assess how much of a sample is "explained" by mappings to reference genomes, we focus on the fraction of reads mapped to the assembly, a metric that was previously used in the literature to assess the "completeness" of metagenomic assemblies.[35,41] The automated reference selection process used by MetaCompass achieved an average 74.2% read mapping rate across the 80 samples we analyzed.

The fraction of reads mapped varied across body sites and different initial numbers of input reads (Figure 5). Notably, samples from the buccal mucosa showed the lowest mean fraction of reads mapped of 51.7%, followed by the anterior nares at 62.4%. These two body sites also exhibit a significantly larger variability of fraction of reads mapped across samples as compared to other body sites. The samples from all other sites exceeded 73.5% mapping rate.

We further explore the relationship between the size of the input, measured as the total number of reads in the sample, and the fraction of reads mapped (Figure 5, top left). For the samples analyzed, MetaCompass achieved high read mapping rates for inputs with more than about 40,000,000 reads. The depth of sequencing needed to reach a fraction of reads mapped exceeding 80% varied across body sites. In the posterior fornix, high mapping rates could be achieved with as few as 4 million reads, while for the buccal mucosa, the threshold could only be reached after 47 million reads. This variability among different body sites can be linked to sample complexity. Specifically, we rely on the sample richness as estimated from the NCBI SRA taxonomy analysis table (number of genomes at higher than 0.01% representation in the sample), shown in Figure 5, top right. The mean richness of posterior fornix samples is 24 (standard deviation: 11.39), while for the buccal mucosa samples it is 129 (standard deviation: 36.41).

Sequencing depth can also explain why certain samples could not be assembled by MetaCompass. The 9 anterior nares samples that failed to recruit reference genomes comprised a median of 463K reads, in contrast with a median of 1.0M reads for the samples that generated assemblies. The buccal mucosa sample that was not assembled has 3.6M reads, whereas the other samples in this body site had a median of 15.4M reads. In comparison to *de novo* assemblies (Figure 5, bottom), MetaCompass achieves lower mapping rates, since we only account for the reads that can be aligned to reference genomes. The difference in mapping rates between MetaCompass and *de novo* approaches varies between simple and/or well-studied communities (such as stool and vaginal samples) and more complex or less well-studied communities (such as oral sites), with the mapping rates for MetaCompass being closer to those of *de novo* methods in the former case.

The effect of database size on assembly coverage

The default database used by MetaCompass includes 251,288 reference genomes from the NCBI RefSeq database, ensuring extensive coverage of bacterial diversity. To evaluate the impact of database size, we constructed an additional database from a much smaller collection of reference genomes, selecting only the genomes tagged as "representative" or "reference" from RefSeq. These typically represent one genome sequence per species that is determined by the RefSeq curators to best represent the species and are typically assembled to a high quality and contiguity. After excluding the reference genomes that are missing genomic sequence files, this database, referred to as the reduced database, includes 11,061 reference genome sequences. Importantly, using the reduced database eliminates the ability of MetaCompass to effectively sample the pangenome of a species.

Using the default database MetaCompass consistently assembles more genomes than with the reduced database (Table S3), a trend observed across a range of body sites and samples. For example, in the tongue dorsum sample NCBI:SRR514250, 211 genomes are assembled using the default database versus 36 with the representative database.

The quality of reference genome sequences can affect assembly quality

When comparing the assembly generated by MetaCompass on the basis of the complete reference database to that based on a reduced database of representative genome sequences

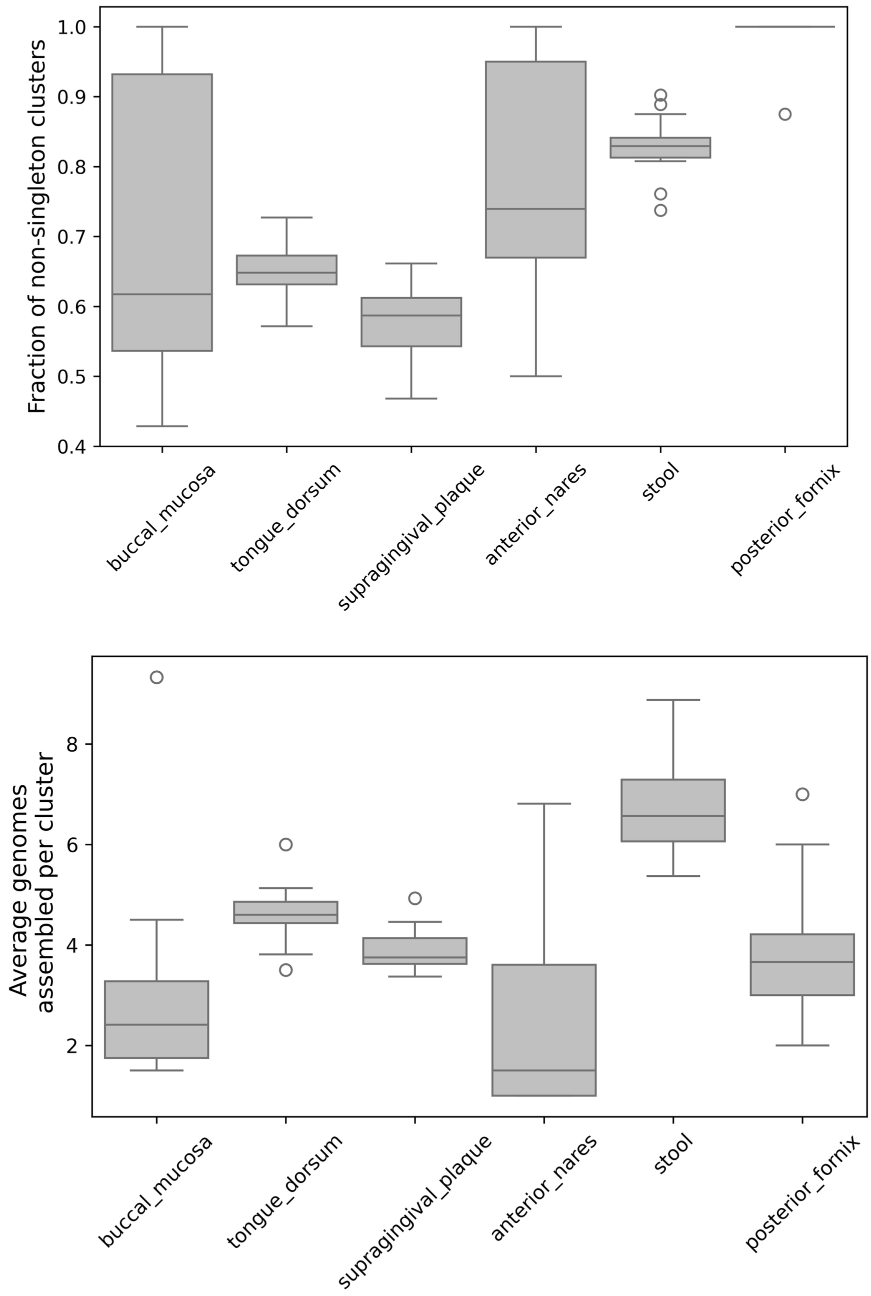

Figure 4. The distribution of non-singleton clusters

Top: fraction of non-singleton clusters per body site. Bottom: number of genomes assembled per non-singleton cluster across body sites. The boxes represent the interquartile range (IQR: 25%–75%); whiskers are extending to the point that is furthest above/below the box that is within 1.5 IQR from the box. Circles represent outliers.

(Table S3), we noted that, for the posterior fornix samples NCBI: SRR513147 and NCBI:SRR628270, the reduced database yielded better assemblies. Specifically, the maximum contig lengths produced by the reduced database were 377K bases and 183K bases for the respective samples compared to 160K bases and 165K bases generated by the default database.

In sample NCBI:SRR513147, the largest assembled contig using the default database was generated based on reference NCBI:GCA_00179995.1, which contains 25 contigs and has an N50 of 92.7K bases. The size of this largest contig, 159K, matched that of the largest contig in the reference genome. In the reduced database, the reference genome used for assembly is NCBI: GCA_001936235.1, which contains a single contig of 1.7M bases, allowing MetaCompass to generate a contig of 377K, over twice the size of the best achievable contig size for the genome reference selected from the default database.

Similarly, for NCBI:SRR628270, the largest assembled contig from the default database is derived from a highly fragmented reference, consisting of 52 contigs with an N50 of 92.4K bases. This contig, measuring 165K bases, matches the length of the segment of the reference genome used to guide the assembly, and a longer contig could be generated when a better-quality reference genome sequence was selected from the reduced database.

Computational performance

We evaluated the runtime performance of MetaCompass on a Linux 16-core server node (3.0 GHz AMD EPYC 7313 processor) with a memory ceiling of 256 GB. The evaluation was performed using a subset of 6 samples representing different body sites and input sizes.

We assessed the runtime performance of MEGAHIT and metaSPAdes by processing the same samples with identical computing resources as those used for MetaCompass. The findings shown in Table 2 indicate that MetaCompass generally has longer runtime than MEGAHIT. However, its runtime is not significantly longer than that of metaSPAdes, except in the case of the tongue dorsum sample NCBI:SRR514250. This particular sample exhibited much higher microbiome richness compared to other benchmarked samples and used a total of 221 reference genomes in contrast to the second most diverse sample, which only recruited 105 reference genomes. Furthermore, using a reduced database, runtime is substantially improved (albeit at a cost in terms of assembly completeness/representativeness, as described above). For instance, in the case of the tongue dorsum sample NCBI:SRR514250, the sample run on the default database was completed in over 16 h in contrast to just 5 h for the reduced database (Table S4).

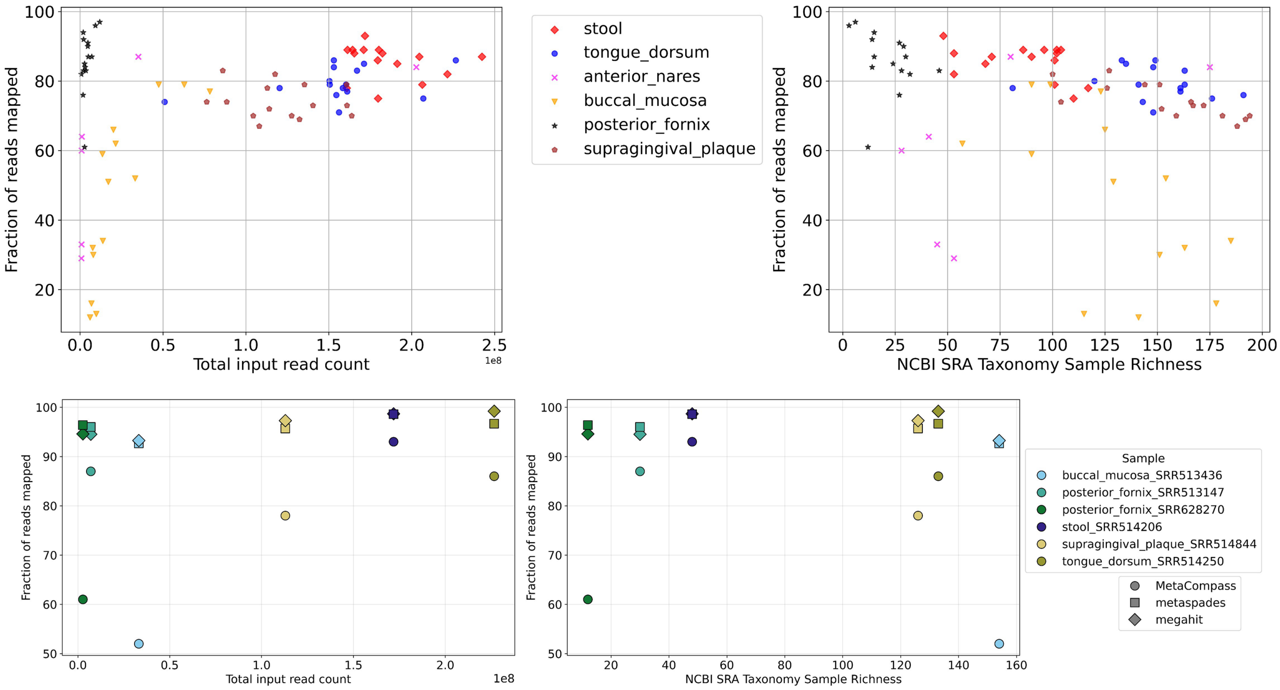

Figure 5. The dependence of read mapping rate on input read count and sample richness

Top: MetaCompass results on the entire dataset. Bottom: comparison with *de novo* methods on six samples with diverse characteristics. Left: total read count. Right: sample richness. Broadly, read mapping rates increase with the total sequencing effort and decrease with sample richness. Mapping rate is typically higher for *de novo* methods, since the MetaCompass assemblies only capture the reads that can be mapped to reference genome sequences.

DISCUSSION

The goal of MetaCompass is to enable reference-guided assembly of metagenomic data in order to leverage the substantial collection of genomic sequences currently available in public databases. As we demonstrate here, the MetaCompass assemblies of individual organisms within metagenomic samples generally outperform *de novo* assemblies of the same organisms. In other words, when appropriate reference genomes are available in the database, MetaCompass is an effective tool for reconstructing MAGs. It is important to note that reference-guided assembly of MAGs has advantages over *de novo* methods, since the gene annotations and other information associated with the reference genome(s) used to guide the assembly are directly associated with the assembly without the need for further computation. Given the broad range of approaches used to construct MAGs through genome binning, we only compared the results of MetaCompass to *de novo* assemblers, showing that the runtime of MetaCompass does not substantially exceed that of commonly used assembly tools. Since *de novo* assembly is just one of the steps used in *de novo* MAG construction, we believe that MetaCompass may match or improve upon the performance of MAG construction pipelines. Importantly, unlike many binning algorithms, MetaCompass can generate high-quality sequences without the need to analyze data from multiple samples.

The effectiveness of reference-based assembly, as implemented by MetaCompass, is significantly affected by the availability and relevance of reference genomes in the reference database being used. In vaginal samples, we identified several genome clusters corresponding to important members of the human vaginal microbiota, where *de novo* assemblies were significantly better than the reference-guided assembly due to differences between the reference genome used by MetaCompass and the data in the sample, suggesting that current databases do not adequately capture the genomic diversity of key members of the vaginal microbiota. Furthermore, the quality and contiguity of reference genomes within public databases significantly impact the performance of reference-based assembly methods, since the quality of the reference sequence is an upper bound on the quality that can be achieved by MetaCompass. This observation suggests the need to carefully curate the database used by MetaCompass (here we used the entirety of RefSeq) to balance taxonomic diversity with reference genome sequence quality. More importantly, for reference-guided analyses to be effective, it is critical that the community continue to invest efforts in sequencing, assembling, and carefully curating bacterial genomes.

We have not explored here the integration between *de novo* assembly and the reference-guided approach used by MetaCompass. Superficially, such integration can be achieved by performing a *de novo* assembly of the reads that are not used by the reference-guided assembly (and MetaCompass provides a utility to do so). Such an approach, however, ignores the opportunity to integrate the two strategies in a non-trivial manner, which requires further research that goes beyond the

CellPress
OPEN ACCESS

Table 2. Assembly statistics and runtime comparison of MetaCompass, MetaSPAdes, and MEGAHIT, evaluated on a Linux 16-core 3.0 GHz AMD EPYC 7313 server with 256 GB memory

Body site	Sample	MetaCompass runtime (h:m:s)	metaSPAdes runtime (h:m:s)	MEGAHIT runtime (h:m:s)	Number of reads	Number of MetaCompass reference genomes assembled
Tongue dorsum	NCBI:SRR514250	16:29:48	07:56:42	01:01:57	226,602,332	211
Buccal mucosa	NCBI:SRR513436	01:09:50	01:19:10	00:16:48	33,183,142	52
Posterior fornix	NCBI:SRR513147	00:21:02	00:14:47	00:02:00	7,038,812	32
Posterior fornix	NCBI:SRR628270	00:07:02	00:05:26	00:00:50	2,713,906	4
Supragingival plaque	NCBI:SRR514844	06:50:27	06:12:57	00:36:37	112,950,078	115
Stool	NCBI:SRR514206	07:56:40	06:03:05	00:24:42	171,825,210	104

scope of this manuscript. Also, a topic of future research is an evaluation of whether the reference-guided strategy used by MetaCompass can provide an advantage over the already effective assembly tools used with long-read data (e.g., Oxford Nanopore or Pacific Biosciences), which are increasingly being used in metagenomic applications. Another opportunity for future research is the integration into MetaCompass of haplotyping algorithms, such as Floria,[42] which would enable a finer analysis of the bacterial strains represented in reference genome cluster.

Limitations of the study

Our ability to compare the quality of the assembly produced by MetaCompass with that produced by *de novo* methods is limited by the nature of the algorithm used by MetaCompass. The commonly used metagenomic assembly validation tool MetaQuast[43] relies on comparisons to appropriately selected reference genomes to determine assembly quality. Since MetaCompass builds the assembly with the help of reference genome sequences, the resulting assembly will have an unfair advantage over *de novo* assemblies when evaluated with MetaQuast. Another approach for validation relies on the consistency of alignments of reads against the assembly[44–46]; however, MetaCompass breaks up the assembly when inconsistencies in read alignment are detected, again favoring MetaCompass over *de novo* methods. Thus, a more thorough evaluation of the relative quality of reference-guided and *de novo* assembly methods is needed but is beyond the scope of this manuscript.

RESOURCE AVAILABILITY

Lead contact

Requests for further information, resources, and reagents should be directed to and will be fulfilled by the lead contact, Mihai Pop (mpop@umd.edu).

Materials availability

This study did not generate new materials.

Data and code availability

- Metacompass assembly outputs are available at https://obj.umiacs.umd.edu/metacompass-assemblies/index.html.
- Our software is released at https://github.com/marbl/MetaCompass under the Artistic License 2.0. The version of the software used in this manuscript is available at Zenodo (https://doi.org/10.5281/zenodo.16876613).
- Any additional information required to reanalyze the data reported in this paper is available from the lead contact upon request.

ACKNOWLEDGMENTS

The authors were supported in part by NIH grants R01-HG-004885 and R01-AI-100947, NSF grants IIS-1117247 and IIS-0812111, and the Office of Naval Research under cooperative agreement number N00173162C001 (all to M.P.). S.K. was supported by the Intramural Research Program of the National Human Genome Research Institute, National Institutes of Health. The content is solely the responsibility of the authors and does not necessarily represent the official views of the National Institutes of Health.

AUTHOR CONTRIBUTIONS

Conceptualization, T.L., B.L., and M.P.; software, T.L., V.P.C.-E., B.L., Z.B., U.A., and T.J.T.; investigation, T.L., V.P.C.-E., T.J.T., S.K., M.A., M.P., and A.P.; validation, T.L., T.J.T., V.P.C.-E., and M.A.; writing – original draft, T.L., V.P.C.-E., M.P., B.L., and T.J.T.; writing – review and editing, all authors. All authors read and approved the manuscript.

DECLARATION OF INTERESTS

The authors declare no competing interests.

STAR★METHODS

Detailed methods are provided in the online version of this paper and include the following:

- KEY RESOURCES TABLE
- METHOD DETAILS
  - Methods overview
  - Reference databases
  - Reference selection
  - Reference culling
  - Cluster-based assembly
  - Contig assembly
  - Contig polishing
  - MetaCompass output
  - Additional software used
- QUANTIFICATION AND STATISTICAL ANALYSIS

SUPPLEMENTAL INFORMATION

Supplemental information can be found online at https://doi.org/10.1016/j.crmeth.2025.101186.

Received: July 15, 2024
Revised: December 16, 2024
Accepted: September 4, 2025
Published: September 26, 2025

REFERENCES

1. Hooper, L.V., and Gordon, J.I. (2001). Commensal Host-Bacterial Relationships in the Gut. Science *292*, 1115–1118. https://doi.org/10.1126/science.1058709.
2. Tringe, S.G., and Rubin, E.M. (2005). Metagenomics: DNA sequencing of environmental samples. Nat. Rev. Genet. *6*, 805–814. https://doi.org/10.1038/nrg1709.
3. Qin, J., Li, R., Raes, J., Arumugam, M., Burgdorf, K.S., Manichanh, C., Nielsen, T., Pons, N., Levenez, F., Yamada, T., et al. (2010). A human gut microbial gene catalogue established by metagenomic sequencing. Nature *464*, 59–65. https://doi.org/10.1038/nature08821.
4. Sunagawa, S., Acinas, S.G., Bork, P., Bowler, C., Tara Oceans Coordinators; Eveillard, D., Gorsky, G., Guidi, L., Iudicone, D., Karsenti, E., et al. (2020). Tara Oceans: towards global ocean ecosystems biology. Nat. Rev. Microbiol. *18*, 428–445. https://doi.org/10.1038/s41579-020-0364-5.
5. Human Microbiome Project Consortium (2012). A framework for human microbiome research. Nature *486*, 215–221. https://doi.org/10.1038/nature11209.
6. McDonald, D., Hyde, E., Debelius, J.W., Morton, J.T., Gonzalez, A., Ackermann, G., Aksenov, A.A., Behsaz, B., Brennan, C., Chen, Y., et al. (2018). American Gut: an Open Platform for Citizen Science Microbiome Research. mSystems *3*, e00031-18. https://doi.org/10.1128/mSystems.00031-18.
7. Kingsford, C., Schatz, M.C., and Pop, M. (2010). Assembly complexity of prokaryotic genomes using short reads. BMC Bioinf. *11*, 21. https://doi.org/10.1186/1471-2105-11-21.
8. Laserson, J., Jojic, V., and Koller, D. (2011). Genovo: De Novo Assembly for Metagenomes. J. Comput. Biol. *18*, 429–443. https://doi.org/10.1089/cmb.2010.0244.
9. Zerbino, D.R., and Birney, E. (2008). Velvet: Algorithms for de novo short read assembly using de Bruijn graphs. Genome Res. *18*, 821–829. https://doi.org/10.1101/gr.074492.107.
10. Peng, Y., Leung, H.C.M., Yiu, S.M., and Chin, F.Y.L. (2012). IDBA-UD: a de novo assembler for single-cell and metagenomic sequencing data with highly uneven depth. Bioinformatics *28*, 1420–1428. https://doi.org/10.1093/bioinformatics/bts174.
11. Simpson, J.T., Wong, K., Jackman, S.D., Schein, J.E., Jones, S.J.M., and Birol, İ. (2009). ABySS: A parallel assembler for short read sequence data. Genome Res. *19*, 1117–1123. https://doi.org/10.1101/gr.089532.108.
12. Li, R., Zhu, H., Ruan, J., Qian, W., Fang, X., Shi, Z., Li, Y., Li, S., Shan, G., Kristiansen, K., et al. (2010). De novo assembly of human genomes with massively parallel short read sequencing. Genome Res. *20*, 265–272. https://doi.org/10.1101/gr.097261.109.
13. Lin, Y.-Y., Hsieh, C.-H., Chen, J.-H., Lu, X., Kao, J.-H., Chen, P.-J., Chen, D.-S., and Wang, H.-Y. (2017). De novo assembly of highly polymorphic metagenomic data using in situ generated reference sequences and a novel BLAST-based assembly pipeline. BMC Bioinf. *18*, 223. https://doi.org/10.1186/s12859-017-1630-z.
14. Rho, M., Tang, H., and Ye, Y. (2010). FragGeneScan: predicting genes in short and error-prone reads. Nucleic Acids Res. *38*, e191. https://doi.org/10.1093/nar/gkq747.
15. Brady, A., and Salzberg, S.L. (2009). Phymm and PhymmBL: metagenomic phylogenetic classification with interpolated Markov models. Nat. Methods *6*, 673–676. https://doi.org/10.1038/nmeth.1358.
16. Liu, B., Gibbons, T., Ghodsi, M., Treangen, T., and Pop, M. (2011). Accurate and fast estimation of taxonomic profiles from metagenomic shotgun sequences. BMC Genom. *12*, S4. https://doi.org/10.1186/1471-2164-12-S2-S4.
17. Segata, N., Waldron, L., Ballarini, A., Narasimhan, V., Jousson, O., and Huttenhower, C. (2012). Metagenomic microbial community profiling using unique clade-specific marker genes. Nat. Methods *9*, 811–814. https://doi.org/10.1038/nmeth.2066.
18. Abubucker, S., Segata, N., Goll, J., Schubert, A.M., Izard, J., Cantarel, B.L., Rodriguez-Mueller, B., Zucker, J., Thiagarajan, M., Henrissat, B., et al. (2012). Metabolic Reconstruction for Metagenomic Data and Its Application to the Human Microbiome. PLoS Comput. Biol. *8*, e1002358. https://doi.org/10.1371/journal.pcbi.1002358.
19. Menzel, P., Ng, K.L., and Krogh, A. (2016). Fast and sensitive taxonomic classification for metagenomics with Kaiju. Nat. Commun. *7*, 11257. https://doi.org/10.1038/ncomms11257.
20. Kolmogorov, M., Bickhart, D.M., Behsaz, B., Gurevich, A., Rayko, M., Shin, S.B., Kuhn, K., Yuan, J., Polevikov, E., Smith, T.P.L., and Pevzner, P.A. (2020). metaFlye: scalable long-read metagenome assembly using repeat graphs. Nat. Methods *17*, 1103–1110. https://doi.org/10.1038/s41592-020-00971-x.
21. Feng, X., Cheng, H., Portik, D., and Li, H. (2022). Metagenome assembly of high-fidelity long reads with hifiasm-meta. Nat. Methods *19*, 671–674. https://doi.org/10.1038/s41592-022-01478-3.
22. Chin, C.-S., Alexander, D.H., Marks, P., Klammer, A.A., Drake, J., Heiner, C., Clum, A., Copeland, A., Huddleston, J., Eichler, E.E., et al. (2013). Nonhybrid, finished microbial genome assemblies from long-read SMRT sequencing data. Nat. Methods *10*, 563–569. https://doi.org/10.1038/nmeth.2474.
23. Koren, S., Harhay, G.P., Smith, T.P.L., Bono, J.L., Harhay, D.M., Mcvey, S.D., Radune, D., Bergman, N.H., and Phillippy, A.M. (2013). Reducing assembly complexity of microbial genomes with single-molecule sequencing. Genome Biol. *14*, R101. https://doi.org/10.1186/gb-2013-14-9-r101.
24. Koren, S., and Phillippy, A.M. (2015). One chromosome, one contig: complete microbial genomes from long-read sequencing and assembly. Curr. Opin. Microbiol. *23*, 110–120. https://doi.org/10.1016/j.mib.2014.11.014.
25. Frank, J.A., Pan, Y., Tooming-Klunderud, A., Eijsink, V.G.H., McHardy, A.C., Nederbragt, A.J., and Pope, P.B. (2016). Improved metagenome assemblies and taxonomic binning using long-read circular consensus sequence data. Sci. Rep. *6*, 25373. https://doi.org/10.1038/srep25373.
26. Driscoll, C.B., Otten, T.G., Brown, N.M., and Dreher, T.W. (2017). Towards long-read metagenomics: complete assembly of three novel genomes from bacteria dependent on a diazotrophic cyanobacterium in a freshwater lake co-culture. Stand. Genomic Sci. *12*, 9. https://doi.org/10.1186/s40793-017-0224-8.
27. Husemann, P., and Stoye, J. (2010). r2cat: synteny plots and comparative assembly. Bioinformatics *26*, 570–571. https://doi.org/10.1093/bioinformatics/btp690.
28. Pop, M., Phillippy, A., Delcher, A.L., and Salzberg, S.L. (2004). Comparative genome assembly. Brief. Bioinform. *5*, 237–248. https://doi.org/10.1093/bib/5.3.237.
29. Timme, R.E., Rand, H., Sanchez Leon, M., Hoffmann, M., Strain, E., Allard, M., Roberson, D., and Baugher, J.D. (2018). GenomeTrakr proficiency testing for foodborne pathogen surveillance: an exercise from 2015. Microb. Genom. *4*, e000185. https://doi.org/10.1099/mgen.0.000185.
30. US FDA GenomeTrakr Network. https://www.fda.gov/food/whole-genome-sequencing-wgs-program/genometrakr-network.
31. Bankevich, A., Nurk, S., Antipov, D., Gurevich, A.A., Dvorkin, M., Kulikov, A.S., Lesin, V.M., Nikolenko, S.I., Pham, S., Prjibelski, A.D., et al. (2012). SPAdes: A New Genome Assembly Algorithm and Its Applications to Single-Cell Sequencing. J. Comput. Biol. *19*, 455–477. https://doi.org/10.1089/cmb.2012.0021.
32. Nurk, S., Bankevich, A., Antipov, D., Gurevich, A., Korobeynikov, A., Lapidus, A., Prjibelsky, A., Pyshkin, A., Sirotkin, A., Sirotkin, Y., et al. (2013). Assembling Genomes and Mini-metagenomes from Highly Chimeric Reads. In Research in Computational Molecular Biology Lecture Notes in Computer Science, M. Deng, R. Jiang, F. Sun, and X. Zhang, eds. (Springer Berlin Heidelberg), pp. 158–170.
33. Prjibelski, A., Antipov, D., Meleshko, D., Lapidus, A., and Korobeynikov, A. (2020). Using SPAdes De Novo Assembler. Curr. Protoc. Bioinforma *70*, e102. https://doi.org/10.1002/cpbi.102.

34. Truong, D.T., Tett, A., Pasolli, E., Huttenhower, C., and Segata, N. (2017). Microbial strain-level population structure and genetic diversity from metagenomes. Genome Res. *27*, 626–638. https://doi.org/10.1101/gr.216242.116.

35. Nurk, S., Meleshko, D., Korobeynikov, A., and Pevzner, P.A. (2017). metaSPAdes: a new versatile metagenomic assembler. Genome Res. *27*, 824–834. https://doi.org/10.1101/gr.213959.116.

36. Li, D., Liu, C.-M., Luo, R., Sadakane, K., and Lam, T.-W. (2015). MEGAHIT: an ultra-fast single-node solution for large and complex metagenomics assembly via succinct de Bruijn graph. Bioinformatics *31*, 1674–1676. https://doi.org/10.1093/bioinformatics/btv033.

37. Seong, H.J., Kim, J.J., Kim, T., Ahn, S.J., Rho, M., Lee, K.J., and Sul, W.J. (2024). Recovery of 240 metagenome-assembled genomes from coastal mariculture environments in South Korea. Sci. Data *11*, 902. https://doi.org/10.1038/s41597-024-03769-0.

38. Barcenilla, C., Cobo-Díaz, J.F., De Filippis, F., Valentino, V., Cabrera Rubio, R., O'Neil, D., Mahler De Sanchez, L., Armanini, F., Carlino, N., Blanco-Míguez, A., et al. (2024). Improved sampling and DNA extraction procedures for microbiome analysis in food-processing environments. Nat. Protoc. *19*, 1291–1310. https://doi.org/10.1038/s41596-023-00949-x.

39. Gurevich, A., Saveliev, V., Vyahhi, N., and Tesler, G. (2013). QUAST: quality assessment tool for genome assemblies. Bioinformatics *29*, 1072–1075. https://doi.org/10.1093/bioinformatics/btt086.

40. Mashima, I., Liao, Y.-C., Lin, C.-H., Nakazawa, F., Haase, E.M., Kiyoura, Y., and Scannapieco, F.A. (2021). Comparative Pan-Genome Analysis of Oral Veillonella Species. Microorganisms *9*, 1775. https://doi.org/10.3390/microorganisms9081775.

41. Schloissnig, S., Arumugam, M., Sunagawa, S., Mitreva, M., Tap, J., Zhu, A., Waller, A., Mende, D.R., Kultima, J.R., Martin, J., et al. (2013). Genomic variation landscape of the human gut microbiome. Nature *493*, 45–50. https://doi.org/10.1038/nature11711.

42. Shaw, J., Gounot, J.-S., Chen, H., Nagarajan, N., and Yu, Y.W. (2024). Floria: fast and accurate strain haplotyping in metagenomes. Bioinforma. Oxf. Engl. *40*, i30–i38. https://doi.org/10.1093/bioinformatics/btae252.

43. Mikheenko, A., Saveliev, V., and Gurevich, A. (2016). MetaQUAST: evaluation of metagenome assemblies. Bioinformatics *32*, 1088–1090. https://doi.org/10.1093/bioinformatics/btv697.

44. Rahman, A., and Pachter, L. (2013). CGAL: computing genome assembly likelihoods. Genome Biol. *14*, R8. https://doi.org/10.1186/gb-2013-14-1-r8.

45. Clark, S.C., Egan, R., Frazier, P.I., and Wang, Z. (2013). ALE: a generic assembly likelihood evaluation framework for assessing the accuracy of genome and metagenome assemblies. Bioinformatics *29*, 435–443. https://doi.org/10.1093/bioinformatics/bts723.

46. Olson, N.D., Treangen, T.J., Hill, C.M., Cepeda-Espinoza, V., Ghurye, J., Koren, S., and Pop, M. (2019). Metagenomic assembly through the lens of validation: recent advances in assessing and improving the quality of genomes assembled from metagenomes. Brief. Bioinform. *20*, 1140–1150. https://doi.org/10.1093/bib/bbx098.

47. Quinlan, A.R., and Hall, I.M. (2010). BEDTools: a flexible suite of utilities for comparing genomic features. Bioinforma. Oxf. Engl. *26*, 841–842. https://doi.org/10.1093/bioinformatics/btq033.

48. Kokot, M., Długosz, M., and Deorowicz, S. (2017). KMC 3: counting and manipulating k -mer statistics. Bioinformatics *33*, 2759–2761. https://doi.org/10.1093/bioinformatics/btx304.

49. Li, H. (2018). Minimap2: pairwise alignment for nucleotide sequences. Bioinformatics *34*, 3094–3100. https://doi.org/10.1093/bioinformatics/bty191.

50. Walker, B.J., Abeel, T., Shea, T., Priest, M., Abouelliel, A., Sakthikumar, S., Cuomo, C.A., Zeng, Q., Wortman, J., Young, S.K., and Earl, A.M. (2014). Pilon: An Integrated Tool for Comprehensive Microbial Variant Detection and Genome Assembly Improvement. PLoS One *9*, e112963. https://doi.org/10.1371/journal.pone.0112963.

51. Li, H., Handsaker, B., Wysoker, A., Fennell, T., Ruan, J., Homer, N., Marth, G., Abecasis, G., and Durbin, R.; 1000 Genome Project Data Processing Subgroup (2009). The Sequence Alignment/Map format and SAMtools. Bioinformatics *25*, 2078–2079. https://doi.org/10.1093/bioinformatics/btp352.

52. Shen, W., Le, S., Li, Y., and Hu, F. (2016). SeqKit: A Cross-Platform and Ultrafast Toolkit for FASTA/Q File Manipulation. PLoS One *11*, e0163962. https://doi.org/10.1371/journal.pone.0163962.

53. Shaw, J., and Yu, Y.W. (2023). Fast and robust metagenomic sequence comparison through sparse chaining with skani. Nat. Methods *20*, 1661–1665. https://doi.org/10.1038/s41592-023-02018-3.

54. Kolmogorov, M., Raney, B., Paten, B., and Pham, S. (2014). Ragout—a reference-assisted assembly tool for bacterial genomes. Bioinformatics *30*, i302–i309. https://doi.org/10.1093/bioinformatics/btu280.

55. Wood, D.E., and Salzberg, S.L. (2014). Kraken: ultrafast metagenomic sequence classification using exact alignments. Genome Biol. *15*, R46. https://doi.org/10.1186/gb-2014-15-3-r46.

56. Li, W., and Godzik, A. (2006). Cd-hit: a fast program for clustering and comparing large sets of protein or nucleotide sequences. Bioinformatics *22*, 1658–1659. https://doi.org/10.1093/bioinformatics/btl158.

57. Jain, C., Rodriguez-R, L.M., Phillippy, A.M., Konstantinidis, K.T., and Aluru, S. (2018). High throughput ANI analysis of 90K prokaryotic genomes reveals clear species boundaries. Nat. Commun. *9*, 5114. https://doi.org/10.1038/s41467-018-07641-9.

58. Chvatal, V. (1979). A Greedy Heuristic for the Set-Covering Problem. Math. Oper. Res. *4*, 233–235. https://doi.org/10.1287/moor.4.3.233.

59. Ondov, B.D., Treangen, T.J., Melsted, P., Mallonee, A.B., Bergman, N.H., Koren, S., and Phillippy, A.M. (2016). Mash: fast genome and metagenome distance estimation using MinHash. Genome Biol. *17*, 132. https://doi.org/10.1186/s13059-016-0997-x.

60. Parks, D.H., Imelfort, M., Skennerton, C.T., Hugenholtz, P., and Tyson, G.W. (2015). CheckM: assessing the quality of microbial genomes recovered from isolates, single cells, and metagenomes. Genome Res. *25*, 1043–1055. https://doi.org/10.1101/gr.186072.114.

61. Chklovski, A., Parks, D.H., Woodcroft, B.J., and Tyson, G.W. (2023). CheckM2: a rapid, scalable and accurate tool for assessing microbial genome quality using machine learning. Nat. Methods *20*, 1203–1212. https://doi.org/10.1038/s41592-023-01940-w.

STAR★METHODS

KEY RESOURCES TABLE

REAGENT or RESOURCE	SOURCE	IDENTIFIER
Deposited data		
Microbiome data from the Human Microbiome Project	NCBI SRA database: https://www.ncbi.nlm.nih.gov/bioproject/PRJNA48479	BioProject:PRJNA48479
Assemblies produced by metacompass	This paper	https://obj.umiacs.umd.edu/metacompass-assemblies/index.html
Software and algorithms		
MetaCompass	This paper	https://github.com/marbl/MetaCompass
Bedtools	Quinlan and Hall[47]	https://github.com/arq5x/bedtools2
KMC	Kokot et al.[48]	https://github.com/refresh-bio/KMC
MEGAHIT	Li et al.[36]	https://github.com/voutcn/megahit
Minimap2	Li[49]	https://github.com/lh3/minimap2
Pilon	Walker et al.[50]	https://github.com/broadinstitute/pilon
Samtools	Li et al.[51]	https://github.com/samtools/samtools
Seqkit	Shen et al.[52]	https://bioinf.shenwei.me/seqkit/
Skani	Shaw and Yu[53]	https://github.com/bluenote-1577/skani

METHOD DETAILS

Methods overview

MetaCompass starts with a collection of genomes that could be used as references (Figure 1). In a reference selection step, we identify a subset of the reference collection representing genomes that could plausibly be used to guide the assembly of the sample being analyzed. We make this determination on the basis of the coverage of universal marker genes —genomes that have a large fraction of the marker genes sufficiently well covered by the reads in the samples are considered further. To address the large extent of redundancy in the reference collection, in a reference culling step, we cluster the refned list of reference genomes based on average nucleotide identity (ANI). During the assembly stage, we proceed on a cluster-by-cluster basis, prioritizing clusters according to the k-mer similarity between each cluster and the read set. To assemble a single cluster, reads are aligned to the references, and contigs are extracted in the order of the extent of coverage for each reference. Consensus sequences are created by "polishing" the reference sequence using Pilon[50] (v1.18). Upon completion, MetaCompass provides assembly statistics and outputs all reads that were not utilized in the assembly process so that they can be easily accessed for additional downstream analyses, such as *de novo* assembly. These individual steps are described in more detail below.

Reference databases

The default database used by MetaCompass consists of the high-quality genome sequences found in the NCBI RefSeq database. Specifically, these are genome sequences that are not flagged as "atypical" or "contaminated", and that are not missing genomic CDS files. The database used to generate the results of this manuscript was constructed in March 2022, and comprised 251,288 genome sequences.

To evaluate the impact of database size on the performance of MetaCompass, we also constructed a reduced database by selecting from RefSeq just those genomes that were marked as "reference" or "representative" in the RefSeq database, i.e., genomes selected by the curators of the database due to their quality and ability to typify microbial species. In this database, we expect only one, or a handful of genomes, per species, leading MetaCompass to create mostly singleton clusters during the reference culling step. This database was constructed in August 2023 and comprised 11,061 genome sequences. The difference in performance between MetaCompass when using the default versus the reduced database is described in Table S3.

Reference selection

While comparative assembly approaches have already been described for single genomes,[28,54] their use in metagenomic data is complicated by the fact that the appropriate reference genome(s) need to be selected in a sample-specific manner from a potentially large collection of genomes (e.g., all publicly available microbial genome sequences). Building efficient indexes for large reference collections is computationally challenging for short-read aligners,[55] both in terms of speed and memory consumption. Therefore, narrowing down the search space for potential reference genomes before initiating any whole genome indexing or read alignment

process is crucial. To tackle this task, we leverage the fact that we are only interested in those genome sequences that will be useful to guide the assembly process, specifically, genome sequences that are sufficiently well covered by the reads in the sample. At a high enough depth of coverage (approx. > 3-fold), we can assume that the vast majority of genes in a genome is covered by reads. Thus, our initial indexing strategy focuses on a relatively small set of universal marker genes, retaining only those genomes that have the majority of these genes covered sufficiently well by the reads in the input.

We use FetchMG[17] to identify 40 universal single-copy marker genes across all reference genomes, in a one-time MetaCompass database-building process. We group sequences from each marker gene into clusters using CD-HIT[56] (version 4.8.1, using command "cd-hit-est -c 0.99 -n 10 -d 0"), with a sequence similarity threshold of 99%. The index construction is a one-time operation, is database-specific, and the index is reused across runs of MetaCompass. During the execution of MetaCompass, all input reads are aligned using Minimap2[49] (version 2.26-r1175, using command minimap2 -ax sr –heap-sort = yes) to the pre-extracted marker gene representative. For a reference genome to be considered for assembly, it must cover at least 75% of the universal marker genes, where a marker gene is considered covered by reads if the breadth of the alignment coverage exceeds 90% of the length of the gene. This step typically narrows down the total number of reference candidates for assembly from 251,288 to a few thousand, yet retains for further analysis genomes that are covered even at low levels of coverage. The effect of depth of coverage on the ability of MetaCompass to detect a sufficient number of marker genes from reference genomes is explored in Table S2.

Reference culling

The reference selection step described above selects a redundant set of genomes. For example, the many *Escherichia coli* genomes available in public databases would be selected if the sample being analyzed contains an *E. coli* strain. Adequately dealing with this ambiguity is critical for effective assembly. If all read mappings are retained, allowing a read to be associated with multiple reference genomes, the resulting assembly will be redundant, reconstructing multiple copies of the homologous genomic regions. If instead, for each read, a random placement is selected from among the multiple equivalent matches, none of the related genomes may recruit enough reads to allow assembly, thereby leading to a fragmented reconstruction. Assigning reads to genomes according to their estimated representation in the sample (determined, e.g., based on the number of reads uniquely mapped to each genome) may bias the reconstruction toward the more divergent reference genomes, which may lead to an overall poorer reconstruction of the genomic regions shared across related genomes.

To address these challenges, we developed a clustering-based approach. We cluster all reference genomes selected from previous steps based on average nucleotide identity (ANI) as estimated by Skani[53] (version 0.2.1, using command 'skani triangle'), using a threshold of 95%—a widely-used cutoff for defining prokaryotic species boundaries.[57] MetaCompass prioritizes the assembly of the clusters on the basis of the overlap between the sequencing reads and the genomic sequences in the cluster. Reads assigned to one cluster are excluded from consideration in the subsequent clusters, thereby avoiding redundancy and reducing workload. The prioritization is accomplished by performing the intersection, using KMC[48] (v3.2.1, using command "kmc -k28 -ci1 -hp -fq"), between the unique k-mer set derived from the genome sequences in the cluster with the k-mers identified from the un-assigned reads. We iteratively process the clusters in order of the size of the k-mer intersection with the reads, as re-computed before each cluster selection, and perform reference-guided assembly by using the reference genomes in the cluster in an iterative approach intended to further restrict the number of genomes processed.

Cluster-based assembly

Genomes within the same ANI-based cluster are closely related and have high genomic similarity, implying that reads are likely to map equally well to multiple reference sequences. To prevent redundancy, we process the genomes in the cluster in order of their overlap with the set of reads, and reads are eliminated from further consideration once used in the assembly of a genome. We use a strategy inspired by the greedy approximation algorithm for the set cover problem[58] by iteratively picking the reference genome to which we can align the majority of the unassigned reads. We are effectively trying to approximate finding the smallest number of reference genomes that "explains" the majority of the reads. The match between a reference genome sequence and the read set is approximated by the k-mer similarity between them using Mash[59] (version 2.3, using the command 'mash screen -w -p 64'). This approach is notably more efficient than direct read-to-genome alignment. Once the genome with the best match to the set of reads is identified, reads are aligned to it and assembled as described in the next section. The reads used in this assembly are removed from further consideration, and the process continues with the un-assembled reads. That is, only reads not used in the assembly of the prior genomes analyzed in the cluster are aligned to the remaining reference genome sequences, in order of their "fit" with the data. The assembly process continues until one of the following criteria is met: (i) all reads are assembled; (ii) all references in the cluster are assembled; (iii) the cumulative length of assembled contigs fails to reach 5% of the reference genome's length; or (iv) the longest contig assembled is shorter than 2,000 base pairs. The final two conditions detect when the process reaches a point of diminishing returns. When the cluster contains a single genome, the assembly is retained even if it fails conditions (iii) and (iv) above.

Contig assembly

The input reads are aligned to the currently-selected reference genome using Minimap2 (version 2.26-r1175, using command minimap2 -ax sr –heap-sort = yes). The output of minimap2 is processed to identify regions of the reference that are not covered by any reads, and contigs are formed by breaking the reference at these locations. We remove from further consideration any contigs thus formed that are shorter than 500 base pairs. We record the locations of contigs within the reference genome and report it in an AGP-formatted file (format version 2.1).

Contig polishing

Our ultimate goal is to reconstruct the sequence of organisms found in the sample rather than simply recapitulating the sequence of the reference genome used as a guide. The process described in the previous paragraph is able to account for major structural differences between the reference sequence and the genome being assembled. To account for nucleotide-level differences between reads and the reference genome, we use Pilon,[50] a widely used polishing tool. This process involves realigning all previously mapped reads to the contigs from the reference-guided assembly using Minimap2, then feeding the resulting BAM file into Pilon for polishing. In addition to the final consensus sequence generated by Pilon for each contig, the full report generated by Pilon is recorded as a part of the MetaCompass output.

MetaCompass output

In addition to a FASTA-formatted file that contains the sequence of all the contigs, MetaCompass also reports the NCBI accessions of the references used in the assembly step. The relative placement of contigs along each reference is reported in an AGP-formatted file, together with the report generated by Pilon. Additionally, MetaCompass outputs all reads that were not used by the reference-guided process, in fastq format, so that they can be fed into subsequent analysis steps, such as *de novo* assembly (and a module is provided to integrate MetaCompass with a *de novo* assembly tool—the Boolean configuration parameter params.skip_denovo can be used to toggle *de novo* assembly on and off).

Additional software used

In addition to software packages already described earlier, MetaCompass and the analysis described here also relied on: bedtools,[47] checkM,[60,61] samtools,[51] and seqkit.[52]

QUANTIFICATION AND STATISTICAL ANALYSIS

To assess the quality of the assemblies produced by MetaCompass and *de novo* assemblers, we relied on several measures that target complementary aspects of the assembly, contiguity, representativeness, completeness, and contamination. **Contiguity** refers to the overall size of the genomic contigs produced by the assembler. In the context of sequence assembly, contiguity is commonly defined in terms of Nx metrics, specifically the largest contig size Nx such that x% of the total assembly size is contained in contigs of size Nx or larger.[39,43] If the size of the genome is known, such metrics are referred to as NGx, indicating that x% of the actual genome size is contained in contigs of size NGx or larger. In our case, the genomes being assembled are known (as defined by the reference genomes selected by MetaCompass) and we used the NG25 and NG50 (25% and 50%, respectively) to compare assemblies in terms of their contiguity. Further details are provided in the Results section. Here we define **representativeness** in terms of the number of reads that can be aligned to the assembly. This measure indicates how much of the information in the data could be used by the assembler. The completeness and contamination are defined by the package CheckM[60,61] as follows. **Completeness** represents an assessment of how much of a genome is present in a genomic bin, as defined by the fraction of a core set of marker genes that are found in the assembly, under the assumption that most bacterial genomes contain the entire set of marker genes. **Contamination** is defined in terms of the number of variants of single copy genes found in the assembly. The presence of multiple variants of a gene typically found in a single copy in most known bacterial genomes indicates that an assembly may contain a mixture of data from more than one genome. While a genome assembler could be tuned to improve one of these metrics at the detriment of others, achieving higher performance according to multiple metrics requires algorithmic improvements.

Cell Reports Methods, Volume *5*

Supplemental information

Reference-guided assembly

# of metagenomes with MetaCompass

Tu Luan, Victoria P. Cepeda-Espinoza, Bo Liu, Zac Bowen, Ujjwal Ayyangar, Mathieu Almeida, Sergey Koren, Todd J. Treangen, Adam Porter, and Mihai Pop

SUPPLEMENTARY MATERIAL

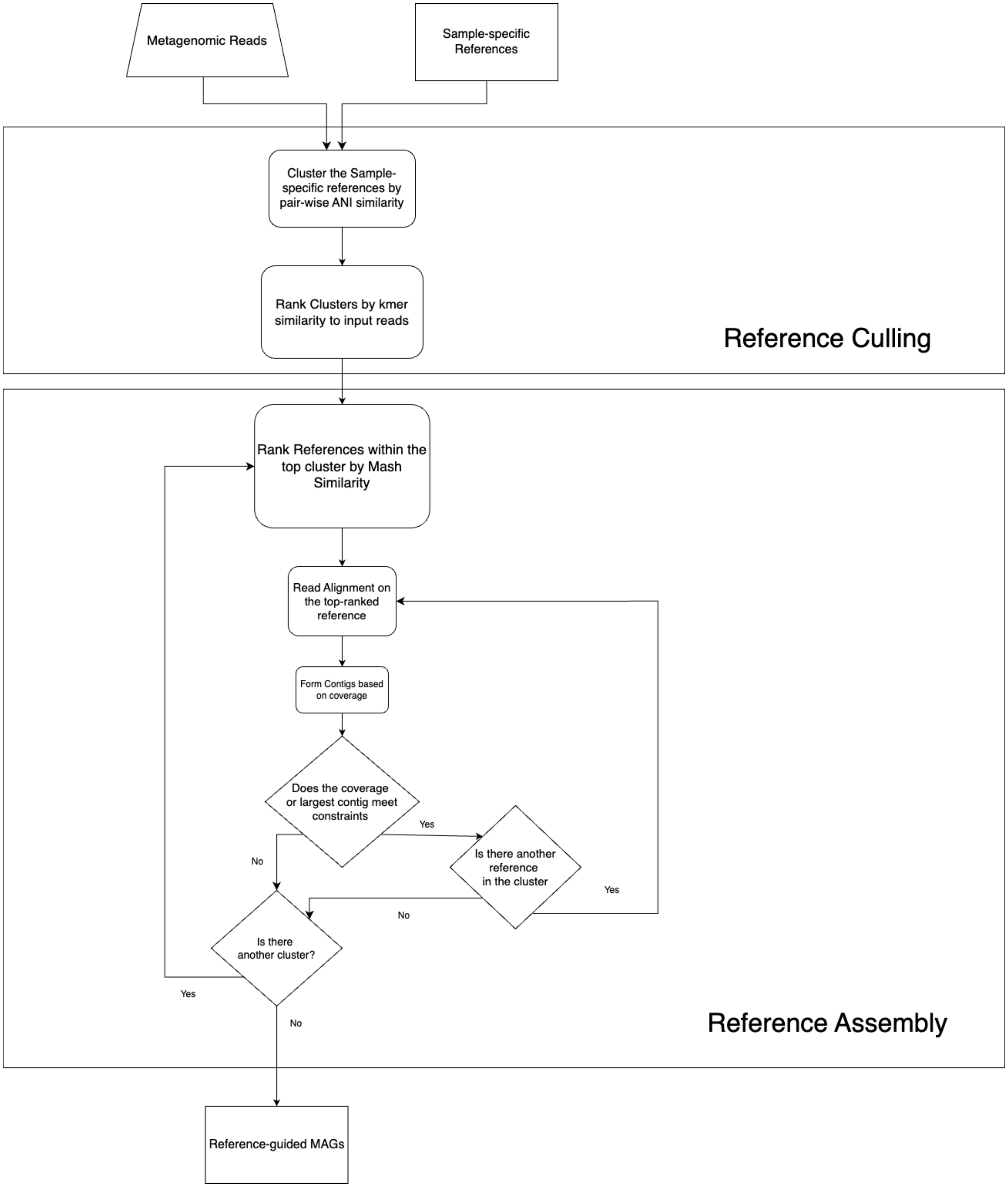

Supplementary Figure S1. The workflow of Reference Selection and Reference Assembly, related to Figure 1.

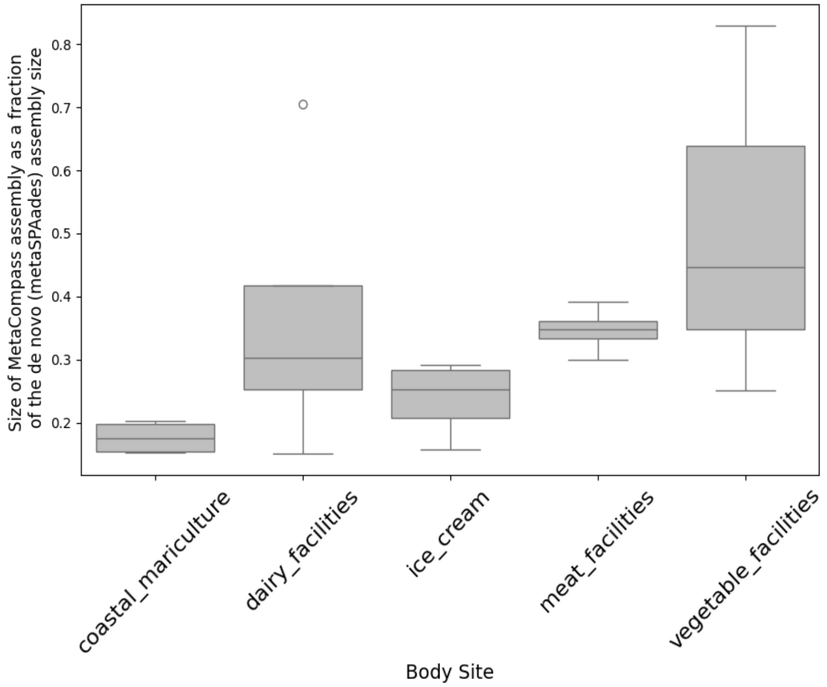

Supplementary Figure S2. Fraction of the de novo assembly that is captured by the reference-guided assembly for non-human samples, related to Figure 2. The numbers are notably lower than those for human-associated microbiome samples, reflecting the poorer representation of environmental microbes in public databases.

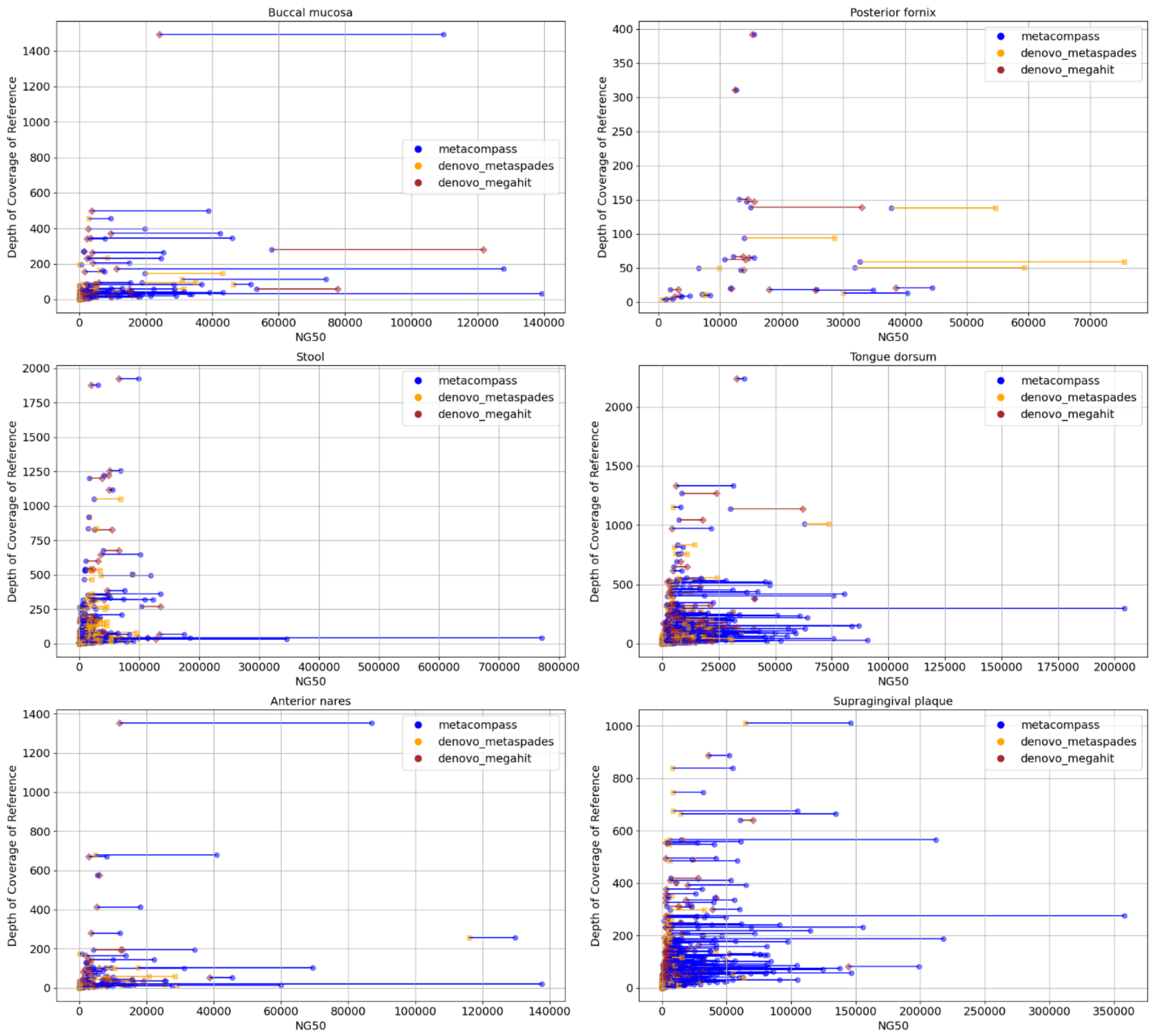

Supplementary Figure S3. Comparison between MetaCompass and *de novo* assembly methods on the measurement of NG50 of the clusters versus the depth of coverage of references, related to Figure 3. Data are shown for 15 stool samples from each body site. The length of the line connecting the two assembly NG50 points represents the difference between the NG50 values of the two points.

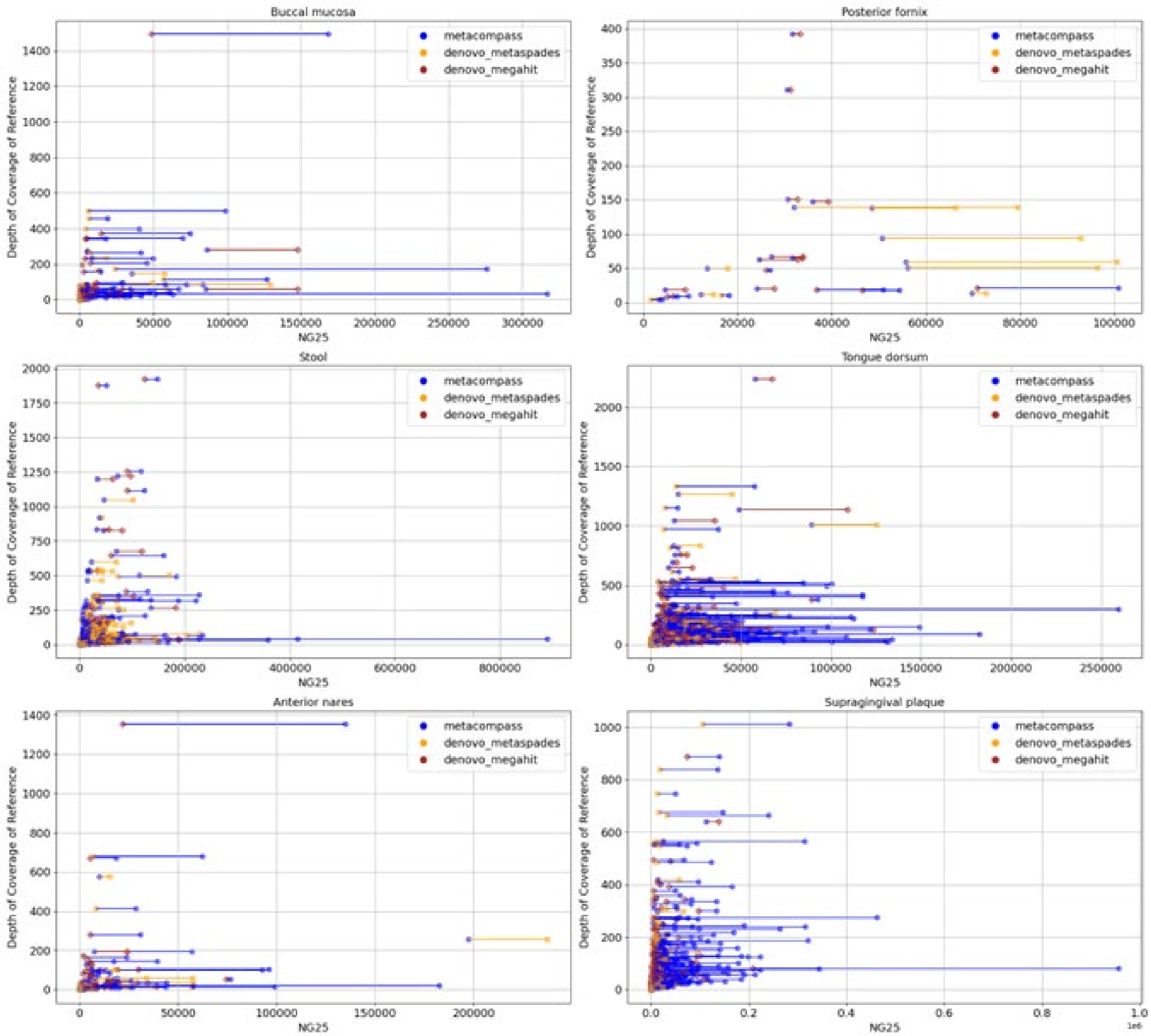

Supplementary Figure S4. Comparison between MetaCompass and *de novo* assembly methods on the measurement of NG25 of the clusters versus the depth of coverage of references, related to Figure 3. Data are shown for 15 samples from each body site. The length of the line connecting the two assembly NG25 points represents the difference between the NG25 values of the two points.

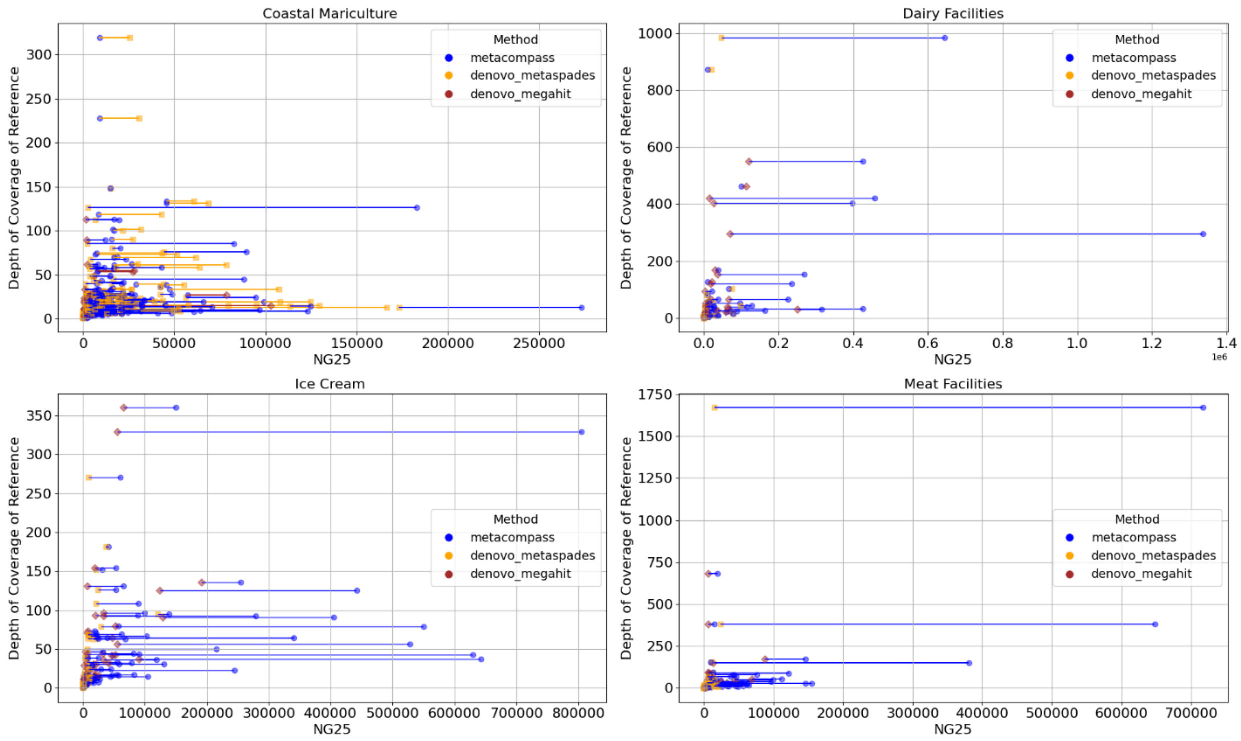

Supplementary Figure S5. Comparison between MetaCompass and *de novo* assembly methods on the measurement of NG50 of the clusters versus the depth of coverage of references for non-human sites, related to Figure 3. The four panels represent: a coastal mariculture system, and sampling of an ice cream manufacturing facility and meat and dairy facilities. The length of the line connecting the two assembly NG25 points represents the difference between the NG25 values of the two points.

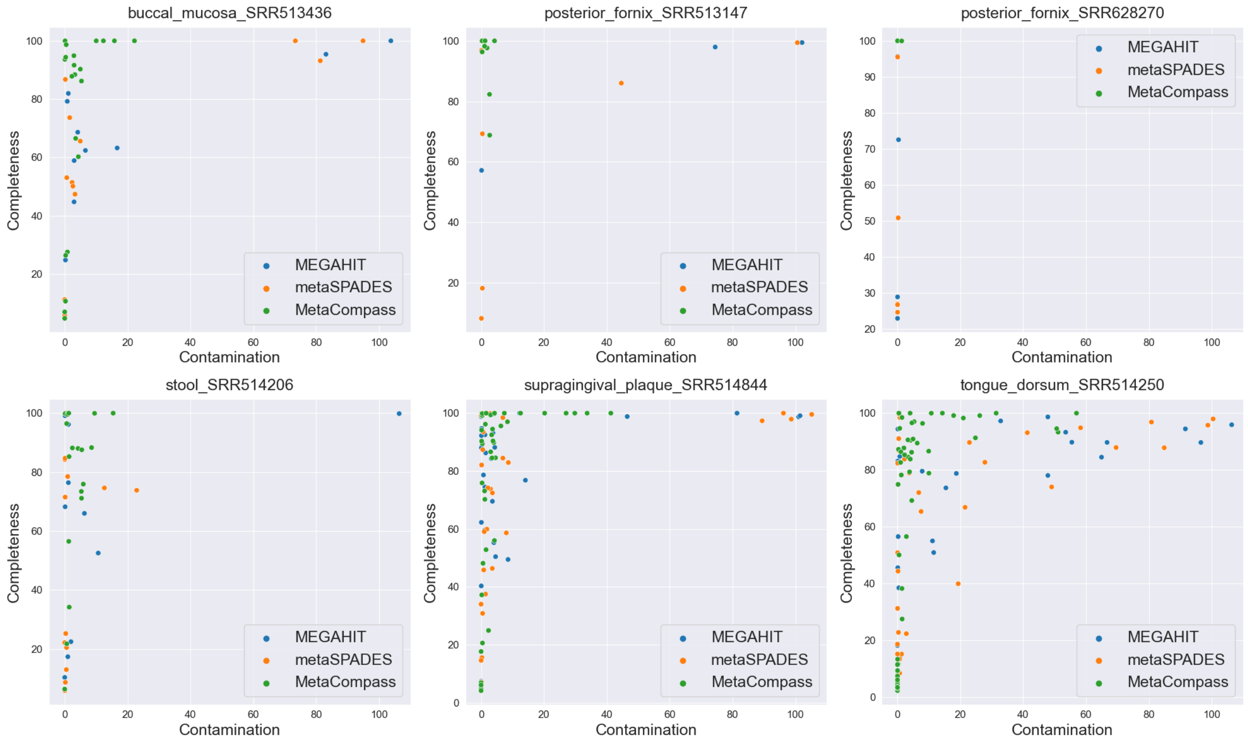

Supplementary Figure S6. Comparison between MetaCompass and *de novo* assembly methods based on completeness and contamination levels for genome-level bins, related to Table 1. Data are shown for six samples from different body sites. MetaCompass bins are defined by the assemblies generated by individual reference genome clusters. *De novo* bins are generated using MetaBat2.

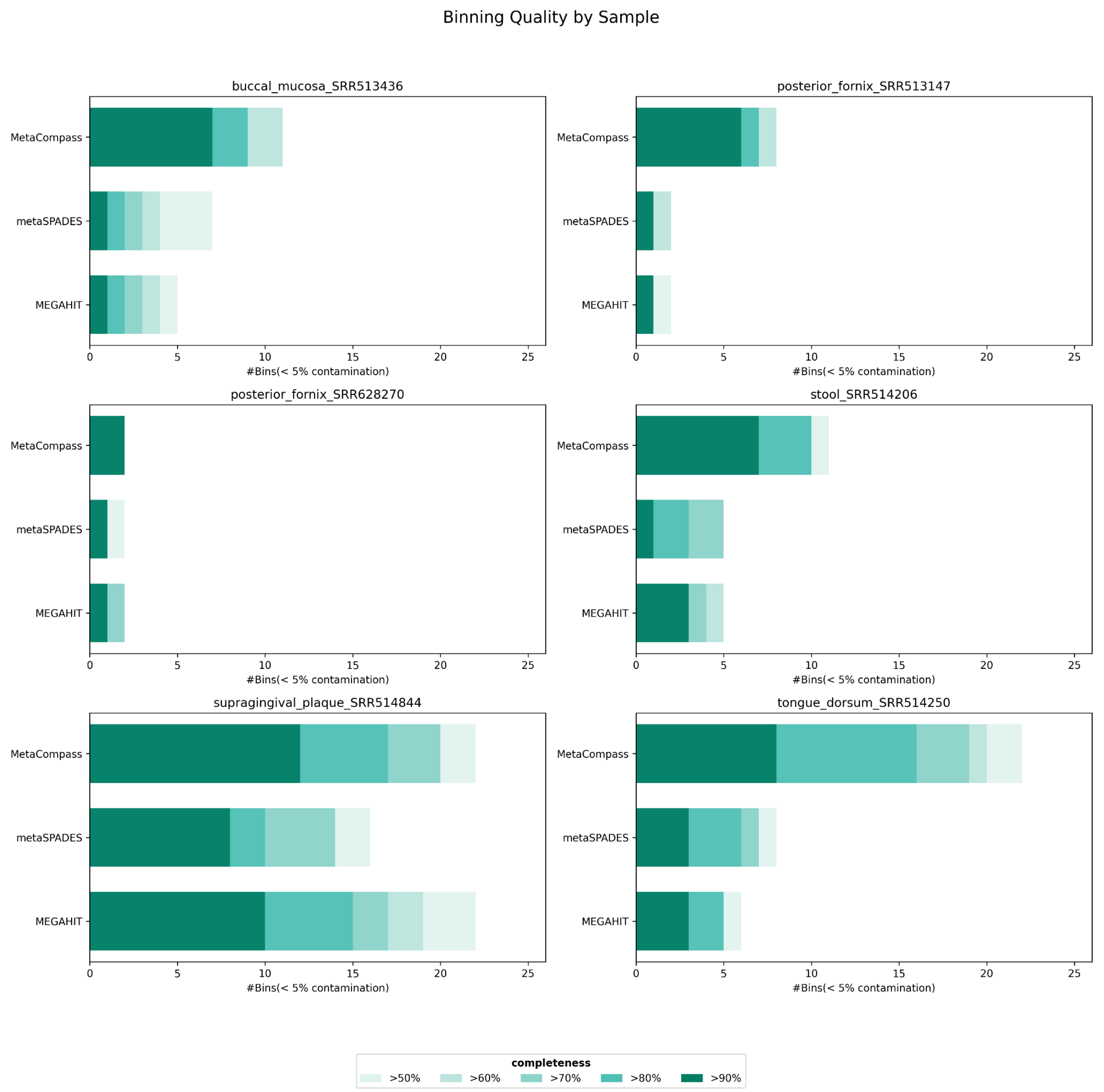

Supplementary Figure S7. Binning completeness for a fixed level of contamination, related to Table 1. A larger number of MetaCompass genome-level bins has a high level of completeness compared to de novo methods, looking only at the bins with low level of contamination.

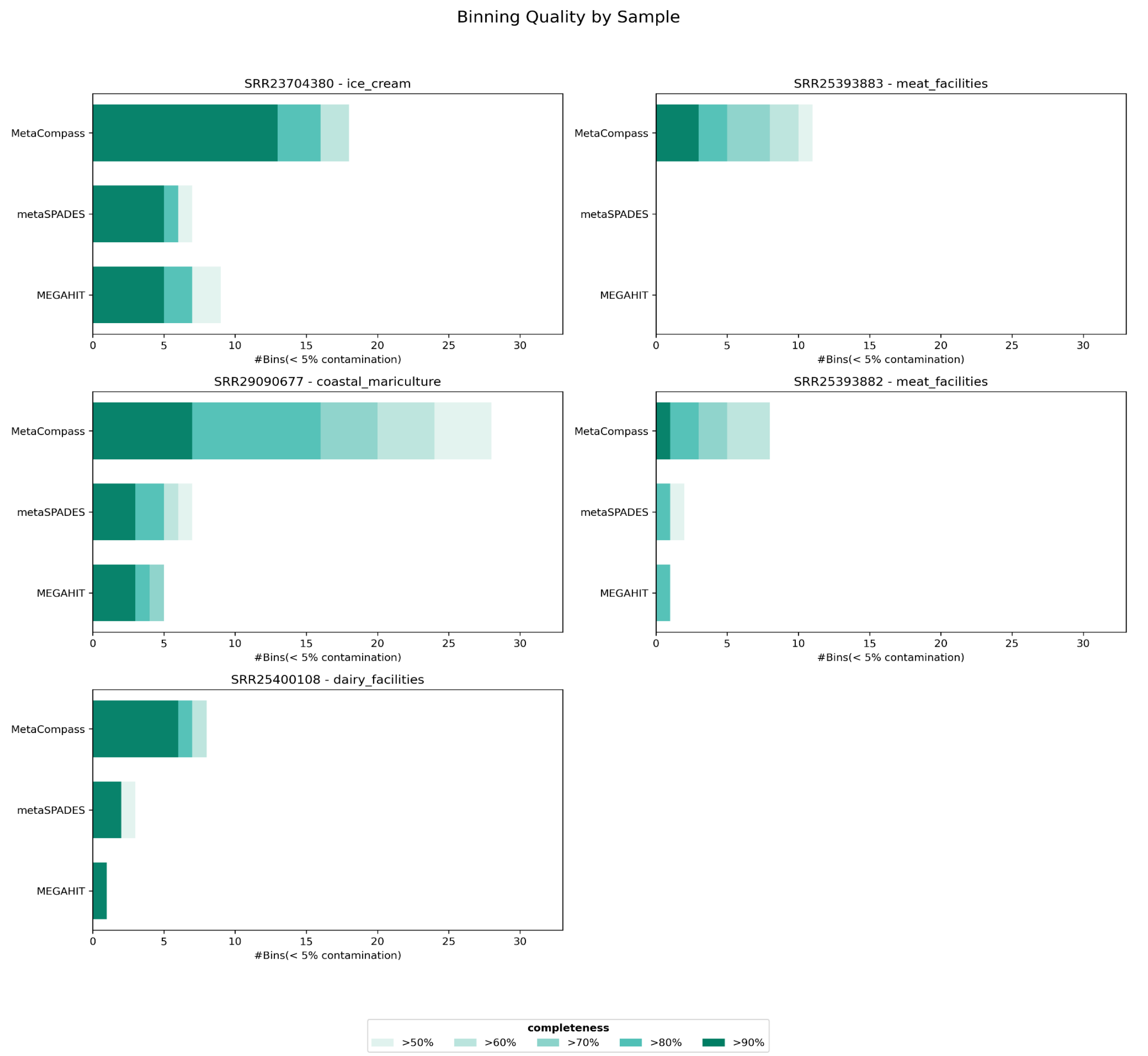

Supplementary Figure S8. Binning completeness versus contamination for non-human microbiome samples, related to Table 1. A larger number of MetaCompass genome-level bins has a high level of completeness compared to de novo methods, looking only at the bins with low level of contamination.

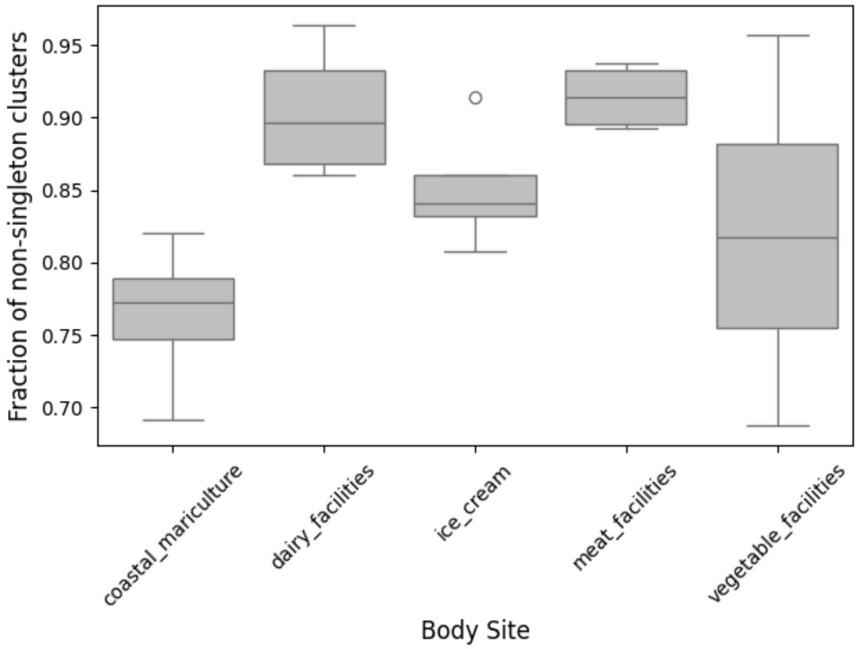

Supplementary Figure S9. Distribution of non-singleton clusters for non-human samples, related to Figure 4. The overall fraction of non-singleton clusters appears slightly higher than for human samples.

Supplementary tables

Supplementary Table S2. Effects of sequencing coverage depth on the reference-guided assembly, Related to STAR Methods. Buccal mucosa sample SRR513142 was analyzed at different levels of rarefaction.

Fraction sub-sampled	Number of reads used by MetaCompass	Number of bases assembled	Average depth of coverage	Total number of marker genes covered in reference selection	Number of reference genomes used
5%	0	0	0	1,470	0
10%	236,390	3,461,370	6.7	1,950	3
20%	704,311	5,682,069	12.2	2,744	4
40%	2,348,667	14,840,897	15.5	3,460	12
60%	3,938,244	22,776,298	17.0	3,846	19
80%	5,436,057	26,890,252	19.8	4,075	22
100%	7,024,805	31,400,140	21.9	4,315	25

Supplementary Table S3. Comparison of assembly statistics between MetaCompass with default database and reduced database, related to STAR Methods.

Body site	Sample	Assembled length using default database (Mbp)	Maximum contig length using default database (kbp)	Assembled length using reduced database (Mbp)	Maximum contig length using reduced database (kbp)
tongue dorsum	SRR514250	95.6	271.3	57.7	180.1
buccal mucosa	SRR513436	38.4	264.4	41.8	127.3
posterior fornix	SRR513147	13.0	159.7	8.8	376.6
posterior fornix	SRR628270	3.4	165.3	6.2	183.3
supragingival plaque	SRR514844	85.4	420.4	70.0	325.6
stool	SRR514206	63.7	406.9	29.1	149.9

Supplementary Table S4. Comparison of runtime and assembly statistics for MetaCompass using the default and reduced databases, related to Table 2. Runtime was evaluated on a Linux 16-core (3.0GHz AMD® EPYC® 7313) server with 256 GB memory.

Body site	Sample	Number of reads	Sample richness	# genomes assembled default database	# genomes assembled reduced database	Runtime default database	Runtime reduced database
tongue dorsum	SRR514250	226,602,332	133	211	36	16:29:48	05:24:49
buccal mucosa	SRR513436	33,183,142	154	52	38	01:09:50	01:15:52
posterior fornix	SRR513147	7,038,812	30	32	6	00:21:02	00:07:48
posterior fornix	SRR628270	2,713,906	12	4	5	00:07:02	00:03:31
supragingival plaque	SRR514844	112,950,078	126	115	45	06:50:27	03:16:16
stool	SRR514206	171,825,210	48	104	14	07:56:40	02:37:06
